# Tryptophan Metabolite Indole‐3‐Aldehyde Induces AhR and c‐MYC Degradation to Promote Tumor Immunogenicity

**DOI:** 10.1002/advs.202409533

**Published:** 2025-06-29

**Authors:** Lei Cui, Zining Wang, Zixuan Guo, Hongxia Zhang, Yongxiang Liu, Huanling Zhang, Huan Jin, Feifei Xu, Xiaojuan Wang, Chunyuan Xie, Hui Guo, Tiantian Wang, Yanxun Lin, Qi Zhao, Penghui Zhou, Jing Tan, Jin‐Xin Bei, Peng Huang, Jinyun Liu, Xiaojun Xia

**Affiliations:** ^1^ State Key Laboratory of Oncology in South China Guangdong Provincial Clinical Research Center for Cancer Sun Yat‐sen University Cancer Center Guangzhou China; ^2^ Department of Pathology School of Basic Medical Sciences Southern Medical University Guangdong Provincial Key Laboratory of Molecular Tumor Pathology Guangzhou China; ^3^ Guangzhou Institute of Clinical Medicine Guangzhou First People's Hospital South China University of Technology Guangzhou China; ^4^ Department of Oncology The Third Affiliated Hospital of Sun Yat‐sen University Guangzhou China; ^5^ Metabolic Innovation Center Zhongshan School of Medicine Sun Yat‐sen University Guangzhou China; ^6^ Hainan Academy of Medical Sciences Hainan Medical University Haikou China

**Keywords:** AhR, c‐Myc, indole‐3‐aldehyde, tryptophan metabolism, tumor immunogenicity

## Abstract

The role of tryptophan (Trp) and its metabolites in immune regulation is well‐established, however, whether and how they regulate immunogenicity of tumor cells is not completely understood. In this study, a range of Trp metabolites are evaluated for their potential to modulate tumor immunogenicity using a co‐culture assay with tumor cells and T cells. Indole‐3‐aldehyde (I3A) is identified as an indole derivative that significantly enhances tumor immunogenicity both in vitro and in vivo. This enhancement is attributed to the upregulation of antigen presentation and immunogenic molecules on tumor cells by I3A, thereby promoting their immunogenicity. Mechanistically, I3A induces the activation and degradation of Aryl hydrocarbon receptor (AhR), leading to increased expression of MHC‐I molecules on tumor cell surfaces. Meantime, I3A induces rapid degradation of c‐MYC in tumor cells and further enhances T cell activation. In mouse melanoma and lymphoma models, I3A demonstrates immune‐dependent antitumor effects and enhances the efficacy of adoptive OT‐I T cell therapy. Moreover, overexpression of the Trp metabolic enzyme interleukin‐4‐induced gene‐1 (IL4I1) in tumor cells increases the intracellular level of I3A and enhances tumor immunogenicity. In summary, I3A is identified as a tumor immunogenicity inducer, which holds the potential to enhance antitumor immunotherapy efficacy.

## Graphical abstract

Tryptophan metabolite Indole‐3‐aldehyde (I3A) downregulates cytosolic AhR and p‐ERK/c‐MYC proteins, together enhancing STAT3 phosphorylation and tumor immunogenicity. Overexpression of the Trp metabolic enzyme interleukin‐4‐induced gene‐1 (IL4I1) in tumor cells increases intracellular levels of I3A and enhances tumor immunogenicity.

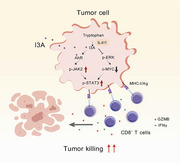

## 1 Introduction

Cancer immunotherapy significantly improves the immune‐deserted tumor microenvironment and has achieved tremendous success.^[^ ^1^ ^]^ The eradication of cancer cells by the immune system primarily occurs through T cell‐mediated recognition and killing of tumors.^[^ ^2^, ^3^ ^]^ T cells recognize tumor antigens bounded with MHC molecules expressed on tumor cells for immune recognition. Thus, tumor antigen expression and its processing on tumor cells determined tumor immunogenicity. Multiple factors including tumor‐intrinsic gene expression and tumor environment may affect tumor immune recognition.^[^ ^1^, ^4^ ^]^ For instance, the release or exposure of damage‐associated molecular patterns (DAMPs) from dying or stressed cells following irradiation or chemotherapy can enhance immune responses by boosting antigen expression and presentation.^[^ ^5^, ^6^, ^7^ ^]^ Interestingly, metabolites derived from tumors or the microbiota may also play a role in modulating tumor immunogenicity.^[^ ^8^, ^9^ ^]^ For example, a recent study found that mitochondrial complex II inhibition enhances tumor immunogenicity by causing succinate accumulation and subsequent epigenetic activation of antigen presentation‐related genes on tumor cells. Furthermore, a mild inhibition of complex II also enhanced immunotherapy efficacy on tumor models, underscoring the potential therapeutic benefits of metabolism‐regulated tumor immunogenicity.^[^ ^8^ ^]^

Besides metabolites from the tricarboxylic acid (TCA) cycle, many amino acids and their metabolites also actively regulate antitumor immunity. Tryptophan (Trp), as an essential amino acid, has been found to play an important metabolic role in multiple cell types in tumor environment.^[^ ^10^, ^11^ ^]^ More than 90% of Trp undergoes enzymatic conversion via indoleamine‐2,3‐dioxygenase 1 (IDO1), IDO2, or Trp‐2,3‐dioxygenase (TDO) to the kynurenine (Kyn) pathway for degradation, resulting in the production of Kyn and subsequent downstream metabolites.^[^ ^12^, ^13^ ^]^ Kyn then activates AhR on tumor cells and neighboring immune cells and induces immune suppression for immune evasion.^[^ ^14^, ^15^, ^16^ ^]^ Blockade of IDO1 via IDO1 inhibitor achieved significant tumor suppression on multiple tumor models, but the clinical trial of IDO1 inhibitor did not show significant efficacy on cancer patients, indicating other unknown mechanisms of Trp metabolism.^[^ ^17^ ^]^ Besides the Kyn pathway, Trp can also be metabolized by multiple bacterial species (such as *Lactobacillus reuteri* present in the intestinal microbiota) to indole and its derivatives indole‐3‐aldehyde (I3A).^[^ ^18^ ^]^ Very recently it was reported that I3A produced by intratumoral bacteria *Lactobacillus reuteri* has an antitumor effect dependent on Tc1 cells,^[^ ^19^ ^]^ and *Lactobacillus gallinarum*‐derived indole‐3‐carboxylic acid (ICA, metabolized from I3A) improved anti‐PD1 efficacy by antagonizing Kyn‐induced Treg differentiation.^[^ ^20^ ^]^ On the contrary, other studies found that tumor or immune cells can also convert Trp to indole pathway via an enzyme IL4I1, which produces indole‐3‐pyruvic acid (I3P) and I3A to activate AhR, ultimately enhancing tumor cell movement, inhibiting T cell proliferation, and correlating with diminished survival rates in individuals with glioma.^[^ ^21^, ^22^, ^23^, ^24^ ^]^ The apparent contradictions in the findings indicate the essential role of Trp metabolites in immune modulation, yet further research is needed to elucidate the specific mechanisms through which these metabolites regulate antitumor immunity differently in various contexts. Additionally, the potential direct impact of these metabolites on tumor cells, particularly in terms of tumor immunogenicity, remains unclear.

In this study, we investigated how Trp metabolites impact tumor immunogenicity via a tumor‐T cell co‐culture system. We found that Trp metabolite I3A treatment rendered tumor cells more immunogenic and facilitated anti‐tumor immune responses. Further investigation found that I3A induced tumor immunogenicity depending on down‐regulation of c‐MYC and AhR, two key transcription factors involved in cancer metabolism, leading to increased expression of MHC‐I and antigen presentation machinery molecules. Our study demonstrated that I3A inhibited tumor growth and potentiated T cell therapy efficacy on mouse melanoma and lymphoma models. Our findings reveal a direct effect of I3A on tumor immunogenicity and may provide a potential means to enhance antitumor immunotherapy efficacy via modulating tumor‐intrinsic Trp metabolism.

## 2 Results

### 2.1 I3A Enhances Tumor Cell‐Induced T Cell Activation

Initially, a tumor‐T cell co‐culture assay was established to assess tumor immunogenicity. It was observed that EG7 cells, which express chicken ovalbumin, were capable of eliciting direct recognition by OVA‐specific T cells and subsequent T cell activation, indicative of tumor immunogenicity. Subsequently, the EG7 tumor cells‐T cells co‐culture system was employed to evaluate the impact of Trp metabolites on tumor immunogenicity. Prior to this investigation, the efficacy of the system was confirmed through the utilization of the known immunogenic drug Teniposide and the immunosuppressive drug Cyclosporin A (CsA).^[^ ^5^, ^25^ ^]^

**Figure 1:**
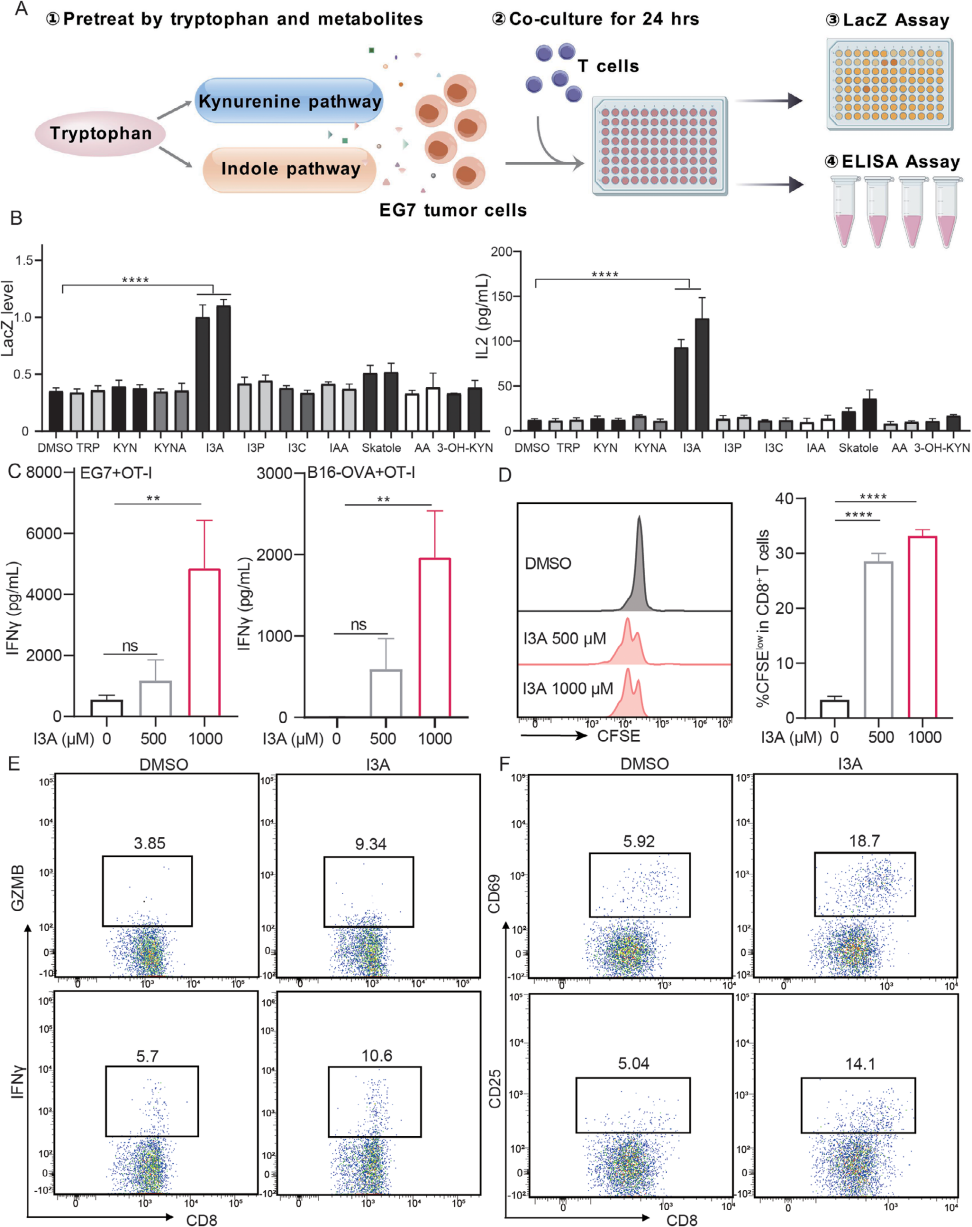
I3A pre‐treated tumor cells induce T cell activation. A) The schematic diagram illustrating the tumor immunogenicity screening assay based on IL‐2 promoter‐driven LacZ reporter. EG7 cells were treated with different metabolites for 18 h, followed by PBS washing and co‐cultured with B3Z T cells in 96‐well plates in a ratio of 1:1 for 24 h, then the OD590 reading value was detected as LacZ reporting activity; B) EG7 cells were treated with tryptophan metabolites (500 µM and 1000 µM) for 18 h, followed by PBS washing and co‐cultured with B3Z T cells for additional 24 h. T cell activation was reflected by IL‐2 driven LacZ activity and IL‐2 ELISA assay. C) EG7 and B16‐OVA cells were treated with I3A (500 and 1000 µm) for 18 h, followed by PBS washing and co‐cultured with naïve OT‐I T cells for 24 h, then the IFNγ production of naïve OT‐I T cells was measured by ELISA. D) EG7 cells were treated with I3A (500 and 1000 µm) for 18 h, followed by co‐culturing with naïve OT‐I T cells labeled with CFSE for an additional 3 days; CFSE signal was detected by FACS. E,F) EG7 cells were pretreated with I3A and then co‐cultured with naïve OT‐I T cells for 24 h, the expression of GZMB, IFNγ, CD25, and CD69 on naïve OT‐I cells was detected by FACS. Bar graphs represent the average ± SEM. *p*‐values were derived from unpaired Student's *t*‐test or one‐way ANOVA analysis of variance with Bonferroni's post‐test for panels B–D. ns, not significant; ** *p* < 0.01; **** *p* < 0.0001. Panel B–F were representative results of at least 3 biological replicates.

We treated EG7 cells with these drugs for a duration of 18 h, removed drugs, and co‐cultured EG7 cells with OVA‐specific CD8^+^ T cell hybridoma B3Z cells for 24 h, then measured IL‐2 promoter–driven LacZ activity and IL‐2 level of the supernatant of co‐culture cells, which reflected IL‐2 expression at mRNA and protein level (**Figure** **1**A). As anticipated, the IL‐2 expression of T cells was markedly enhanced after co‐cultured with tumor cells pre‐treated with Teniposide compared to that of T cells co‐cultured with non‐treated tumor cells, and decreased by CsA pre‐treatment, indicating immunogenicity‐modulating effect of these drugs (Figure S1A, Supporting Information). Subsequently, we examined the impact of various Trp‐derived metabolites on tumor immunogenicity using this co‐culture system (**Table** **1**). Among the Trp metabolites tested, we found that only I3A, an indole metabolite derived from Trp, notably stimulated IL‐2 promoter activity and protein secretion in B3Z T cells (Figure 1B). Consistent with the increased IL‐2 production in B3Z T cells, the supernatant level of cytokine IFNγ significantly increased in primary OT‐I T cells (CD8^+^ T cells that specifically recognize OVA antigen) co‐cultured with EG7 cells pre‐treated with I3A. Furthermore, low immunogenic B16‐OVA cells treated with I3A also activated T cells (Figure 1C), suggesting a powerful immunogenicity‐inducing effect of I3A. Meanwhile, CFSE‐labelled OT‐I cells co‐cultured with I3A‐treated EG7 cells showed higher levels of proliferation (Figure 1D). Consistently, the proportion of naïve OT‐I T cells expressing the activation marker CD25, CD69, and effector molecules IFNγ and GZMB significantly increased after co‐culture with I3A‐treated EG7 cells (Figure 1E,F; Figure S1B,C, Supporting Information). Similarly, the expression levels of CD25, CD69, and effector molecules IFNγ and GZMB were significantly increased in T cells co‐cultured with I3A‐treated B16‐OVA tumor cells (Figure S1D–G, Supporting Information). Taken together, our findings suggest that I3A has the potential to enhance the immunogenicity of tumor cells.

**Table 1:** Tryptophan Metabolites used in the co‐culture screening experiment.

Metabolites	Abbreviate
L‐Tryptophan	TRP
L‐Kynurenine	L‐KYN
Kynurenic acid	KYNA
Anthranilic acid	AA
Indole‐3‐aldehyde (Indole‐3‐carboxaldehyde)	I3A
Indole‐3‐pyruvic acid	I3P
Indole‐3‐carbinol	I3C
Indole‐3‐acetic acid	IAA
3‐Methylindole	Skatole
3‐Hydroxy‐DL‐kynurenine	3‐OH‐KYN

### 2.2 I3A Treatment Induces Immunogenic Markers on Tumor Cells

**Figure 2:**
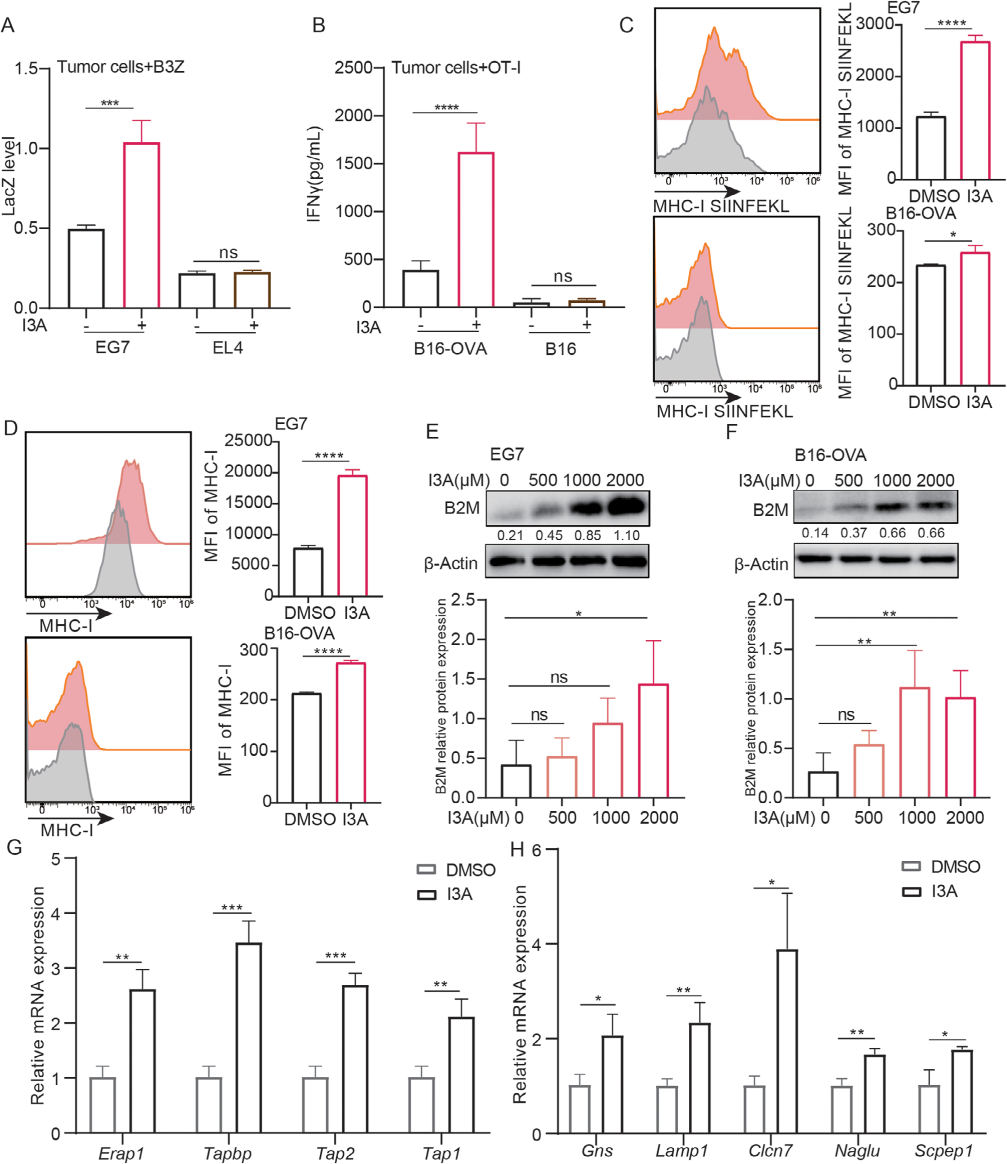
I3A treatment induces immunogenic markers on tumor cells. A) EG7 and EL4 tumor cells were treated with I3A (1000 µm) for 18 h, followed by PBS washing and co‐cultured with B3Z T cells for 24 h. The LacZ level was measured; B) B16‐OVA and B16 tumor cells were treated with I3A for 18 h, followed by PBS washing and co‐cultured with naïve OT‐I T cells for 24 h. The IFNγ level was measured by ELISA assay; C,D) EG7 and B16‐OVA cells were treated with I3A for 18 h, then the expression of MHC‐I and MHC‐I‐SIINFEKL were detected by FACS. The expression of MHC‐I‐SIINFEKL complex on tumor cells was detected by staining with a monoclonal antibody recognizing OVA_257‐264_ (SIINFEKL) peptide bound to H‐2Kb. E,F) EG7 and B16‐OVA cells were treated with I3A for 18 h, then the expression of B2M was detected by WB. G,H) EG7 cells were treated with I3A for 12 h, and the expression of antigen‐presenting machinery genes and lysosome‐related genes were measured by q‐PCR. Bar graphs represent the average ± SEM. *p*‐values were derived from unpaired Student's *t*‐test or one‐way ANOVA analysis of variance with Bonferroni's post‐test for panel A‐H. ns, not significant; *p < 0.05; **p < 0.01; ***p < 0.001; ****p < 0.0001. The results shown were representative results of at least 3 biological replicates.

Previous studies demonstrated that indole derivatives have the ability to directly activate T cells.^[^ ^19^ ^]^ In order to investigate whether residual I3A in tumor culture could potentially activate T cells in our experimental system, we conducted a co‐culture experiment using I3A‐treated EL4 cells or EG7 cells (EL4 cells stably expressing OVA) with B3Z cells for 24 h, followed by evaluation of T cell activation levels. The results indicated that only EG7 cells but not EL4 cells pre‐treated with I3A induced IL‐2 secretion in B3Z T cells (**Figure** **2**A). Consistently, only B16‐OVA cells but not B16 cells pre‐treated by I3A induced IFNγ secretion of naïve OT‐I T cells (Figure 2B). Moreover, naïve OT‐I T cells and B3Z cells treated with I3A directly at indicated concentrations showed no increase of IFNγ level or IL‐2 promoter activity by ELISA and LacZ assay (Figure S2A,B, Supporting Information). Thus, I3A did not directly activate naïve T cells, and T cell activation induced by I3A‐treated tumor cells requires tumor antigen expression in the co‐culture system. As immunogenic cell death facilitates tumor antigen release and potentially antigen presentation, we assessed the viability of tumor cells following treatment with I3A. While I3A treatment did not result in significant cell death, it did significantly impede the proliferation of tumor cells (Figure S2C–E, Supporting Information), leading to alterations in cell morphology characterized by a mesenchymal‐like and elongated spindle shape (Figure S2F, Supporting Information). Nevertheless, we proceeded and treated tumor cells by I3A together with different cell death pathway inhibitors, including apoptosis inhibitor Z‐VAD‐FMK, necroptosis inhibitor Necrostatin‐1, ferroptosis inhibitor ferrostatin‐1, and antioxidant N‐Acetyl‐L‐cysteine (NAC). It was observed that none of these pathway inhibitors hindered the activation of T cells induced by I3A‐treated tumor cells, suggesting that the enhancement of tumor immunogenicity by I3A is not dependent on established cell death pathways (Figure S2G, Supporting Information).

Given the striking tumor immunogenicity elicited by I3A, we checked the expression levels of MHC‐I and MHC‐I‐SIINFEKL complex on I3A‐treated tumor cells. Flow cytometry analysis revealed a significant increase in the expression levels of MHC‐I and MHC‐I‐SIINFEKL on I3A‐treated EG7 and B16‐OVA cells (Figure 2C,D). Furthermore, the expression of B2M, a key subunit of the MHC‐I complex, was also elevated in I3A‐treated tumor cells (Figure 2E,F). And we also assessed the expression levels of a panel of antigen‐presenting machinery genes (*B2m, Erap1, Tapbp, Tap2, Tap1*) and lysosome‐related genes (*Gns, Lamp1, Clcn7, Naglu, Scpep1*) in tumor cells following I3A treatment. Q‐PCR analysis indicated significant upregulation of tumor antigen presentation‐related gene expression levels in response to I3A treatment, potentially contributing to the enhanced tumor antigenicity (Figure 2G,H, Figure S2H,I, Supporting Information). Collectively, these findings suggest that I3A treatment promotes antigen presentation in tumor cells, thereby augmenting tumor immunogenicity.

### 2.3 IL4I1 Catalyzes Tumor‐Intrinsic I3A Production to Induce Tumor Immunogenicity

**Figure 3:**
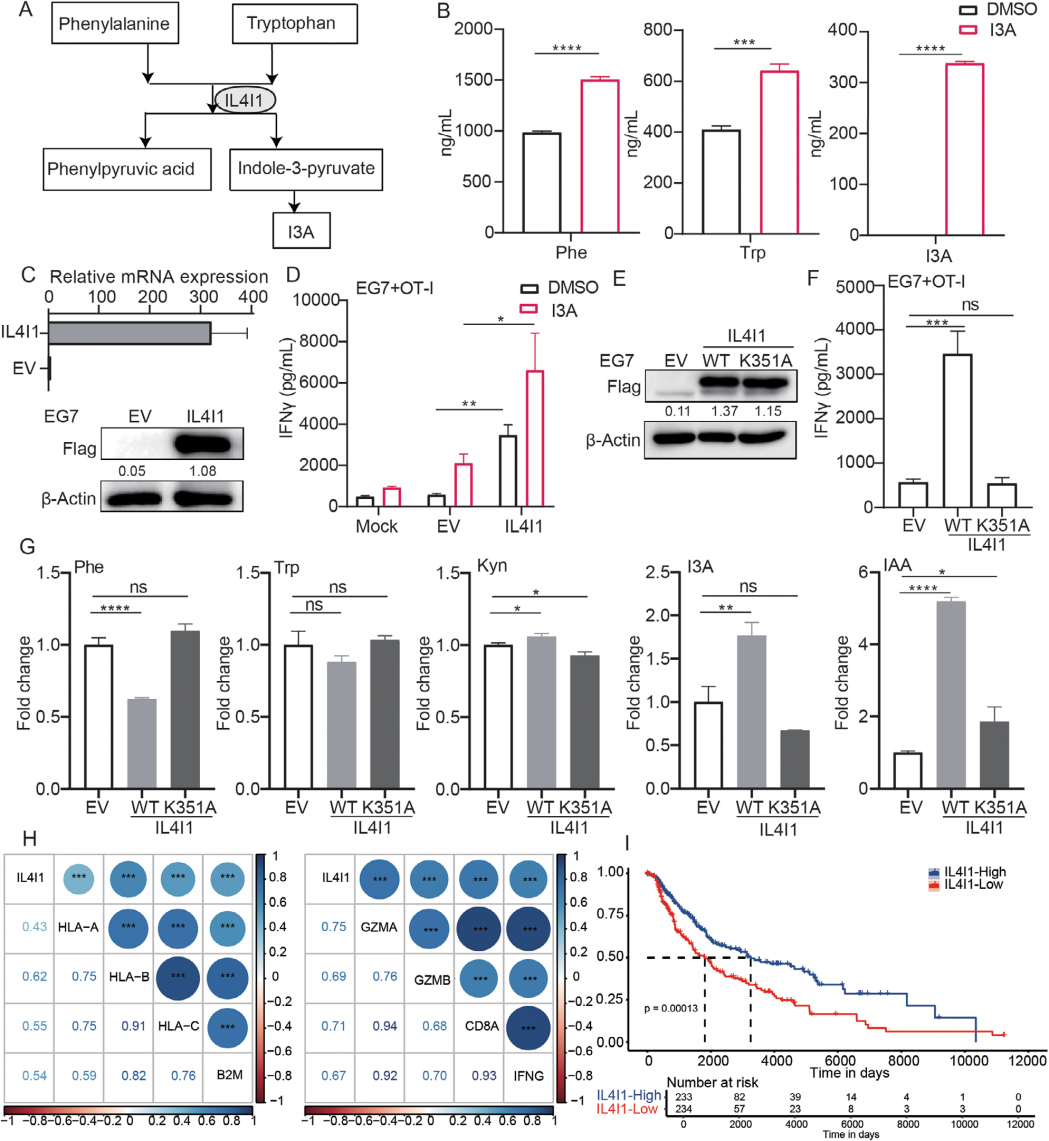
IL4I1 catalyzes tumor‐intrinsic I3A production to induce tumor immunogenicity. A) The enzyme IL4I1 catalyzes tryptophan (Trp) and phenylalanine (Phe) to I3P and phenylpyruvic acid; B) EG7 tumor cells were treated with I3A for 18 h, then the intracellular concentrations of Phe, Trp and I3A in EG7 tumor cells were detected by LC‐MS/MS; C) The EG7 tumor cells which were overexpressed IL4I1 or empty vector (EV), were used for q‐PCR and WB assay to verify the overexpression of IL4I1. D) EG7 tumor cells expressing EV or IL4I1 were treated with I3A for 18 h, followed by PBS washing and co‐cultured with naïve OT‐I cells for 24 h, and the IFNγ level was measured by ELISA assay. E) Protein extracts of EG7 cells overexpressing IL4I1 WT or IL4I1 K351A mutant were collected, and the expression level of IL4I1 was measured by Western blot; F) EG7 tumor cells expressing EV/IL4I1‐WT/IL4I1 K351A were co‐cultured with naïve OT‐I cells for 24 h, then the IFNγ level was measured by ELISA assay. G) The intracellular concentrations of tryptophan metabolites of EG7 cells expressing EV/IL4I1‐WT/IL4I1 K351A mutant were detected by LC‐MS/MS and shown in fold change. H) Correlation of IL4I1 expression with MHC‐I and B2M in SKCM patients and correlation of IL4I1 expression with CTL activation signature (GZMA, GZMB, CD8A, and IFNG) in SKCM patients. I) The correlation of the IL4I1 expression level with the overall survival of SKCM patients, with *p*‐values calculated using the Log‐Rank test. Bar graphs represent the average ± SEM. P values were derived from unpaired Student's *t*‐test or one‐way ANOVA analysis of variance with Bonferroni's post‐test for panel B‐G. ns, not significant; *p < 0.05; **p < 0.01; ***p < 0.001; ****p < 0.0001. The results shown were representative results of at least 3 biological replicates.

Recently it is reported that IL‐4‐Induced Gene‐1 (*Il4i1*) gene, also known as *Fig1* gene, encodes an L‐amino acid oxidase that converts Phenylalanine (Phe), Tyrosine, and Trp to phenylpyruvic acid, hydroxyphenylpyruvic acid (HPP), and indole‐3‐pyruvic acid (I3P), respectively.^[^ ^22^ ^]^ Trp turned to I3P by IL4I1 and subsequently metabolized to I3A (**Figure** **3**A). We performed liquid chromatography‐tandem mass spectrometry (LC‐MS/MS) to detect Trp metabolism in tumor cells, and found I3A treatment induced Trp and Phe accumulation besides I3A in tumor cells (Figure 3B), potentially as a result of a negative feedback loop on Trp metabolism mediated by IL4I1. These findings prompted us to verify whether IL4I1 participated in I3A‐induced tumor immunogenicity. As expected, overexpression of IL4I1 on EG7 cells significantly upregulated T cell‐produced IFNγ and IL‐2 levels, which could be further increased by I3A treatment (Figure 3C,D; Figure S3A, Supporting Information).

To verify whether the T cells activation effect of IL4I1‐expressing tumor cells depends on IL4I1 enzyme activity, we next constructed a K351A mutant, the enzyme‐dead mutant form of IL4I1,^[^ ^26^ ^]^ and overexpressed it on EG7 cells. Western blot analysis revealed that the wild‐type (WT) and K351A mutant forms of IL4I1 were expressed at similar levels in IL4I1‐WT and K351A‐expressing tumor cells (Figure 3E; Figure S3B, Supporting Information). Strikingly, IL4I1‐K351A‐expressing EG7 cells could not boost OT‐I or B3Z T cell activation in tumor‐T co‐culture setting (Figure 3F; Figure S3C, Supporting Information). This result confirmed that IL4I1 overexpression boosts EG7 tumor immunogenicity depending on its enzyme activity. Consistently, LC‐MS/MS results suggested IL4I1‐WT cells had higher levels of I3A but lower levels of Trp and phenylalanine than control EG7 cells, which in accordance with the fact that IL4I1 enzyme is responsible for the metabolism of Trp and Phenylalanine (Figure 3G). We conducted a correlation analysis to examine the relationship between human tumor‐intrinsic expression of IL4I1 and tumor immunogenicity factors such as MHC‐I expression and Cytotoxic T lymphocyte (CTL) activation. Our findings indicate a positive correlation between tumor‐intrinsic IL4I1 expression and the expression of *HLA‐A/‐B/‐C, B2M*, and genes associated with CTL activation (*GZMB, GZMA, CD8A, IFNG*) in melanoma patients (Figure 3H; Figure S3D,E, Supporting Information). Additionally, the expression of IL4I1 in melanoma patients is positively associated with overall survival (Figure 3I).

Collectively, these results suggest that tumor‐intrinsic I3A would enhance tumor immunogenicity, meanwhile IL4I1 overexpression increased tumor immunogenicity by increasing intracellular I3A production.

### 2.4 I3A Inhibits Tumor Growth in a T Cell‐Dependent Manner

**Figure 4:**
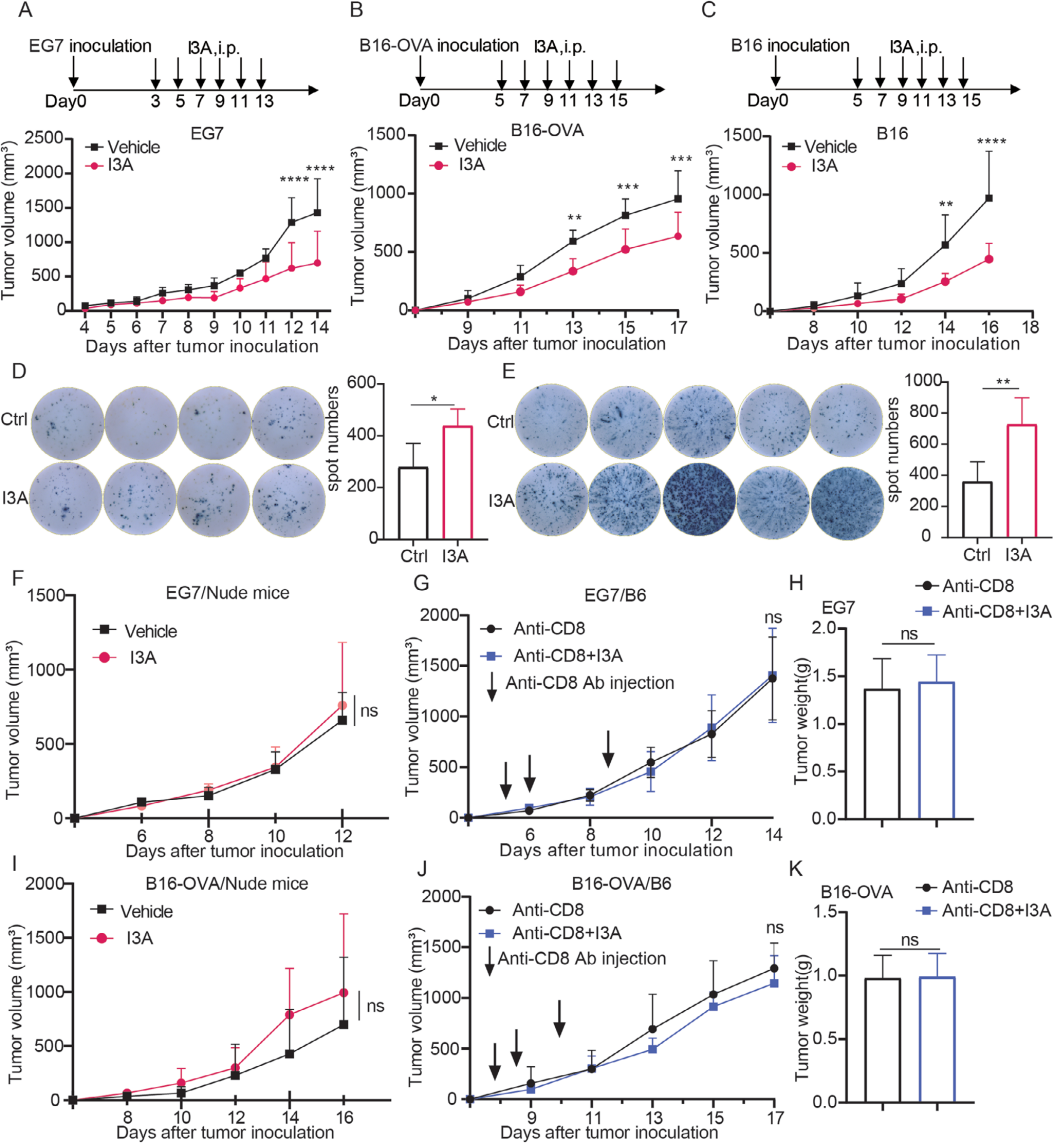
I3A inhibits tumor growth via a T cell‐dependent manner. A–C) Schematic illustration of mouse tumor model for I3A treatment; EG7 cells (1 million per mouse) A), B16‐OVA cells (0.2 million per mouse) B) and B16 cells (0.2 million per mouse) C) were s.c. inoculated in B6 mice, followed by intraperitoneal (i.p.) injection of I3A (50 mg kg^−1^) or vehicle solvent (Ctrl) on day 3 for EG7 tumor or day 5 for B16/B16‐OVA tumor, once every other day, then the tumor growth was recorded. EG7 tumor: n = 6 (Ctrl), n = 7 (I3A); B16‐OVA: n = 6 per group, B16: n = 7 per group; D,E) Mice were i.p. injected with I3A or vehicle from day 5 or day 3 after B16 and EG7 tumor inoculation, and the tumors were harvested on day 15 for tumor‐infiltrating lymphocytes (TILs) isolation for IFNγ ELISpot assay. The TILs from B16 tumors were stimulated by BMDCs primed with B16 tumor lysate D), or stimulated by OVA_257‐264_ peptide for TILs from EG7 tumors E); n = 5 for EG7 tumors and n = 4 for B16 tumors; F) EG7 cells (1 million per mouse) were s.c. inoculated in nude mice, I3A (50 mg kg^−1^) or vehicle were i.p. injected once every other day, then the tumor growth was recorded; n = 5 per group. G,H) EG7 cells (1 million per mouse) were s.c. inoculated in C57 mice, followed by I3A (50 mg kg^−1^) or vehicle treatment (i.p. injection, every other day); anti‐CD8 depletion antibody (100 µg/mouse) were injected at days 3, 6, and 9, then the tumor growth was recorded G), and the tumors were weighed at the end of the experiment H); n = 8 per group. I) B16‐OVA cells (0.2 million per mouse) were s.c. inoculated in nude mice, I3A (50 mg kg^−1^) or vehicle were i.p. injected once every other day, and then the tumor growth was recorded. n = 6 per group. J,K) B16‐OVA cells (0.2 million per mouse) were s.c. inoculated in B6 mice, followed by I3A (50 mg kg^−1^) or vehicle treatment (i.p. injection, every other day); anti‐CD8 depletion antibody was injected at day 4, 7, and 10, then the tumor growth was recorded J) and tumors were weighed at the end of the experiment K). n = 7 for CD8 depletion group, n = 6 for CD8 depletion + I3A group. The tumor weight was recorded in K). Bar graphs represent the average ± SEM. *p*‐Values were derived from unpaired Student's *t*‐test or two‐way ANOVA analysis of variance with Bonferroni's post‐test for panel A‐K. ns, not significant; *p < 0.05; **p < 0.01; ***p < 0.001; ****p < 0.0001.

Subsequently, we investigated the impact of I3A administration on the immune regulation of tumor progression in an in vivo setting. Administration of I3A via intraperitoneal injection resulted in notable suppression of tumor growth in EG7 lymphoma, B16, and B16‐OVA melanoma‐bearing mice (**Figure** **4**A–C; Figure S4A–F, Supporting Information). Immunotyping of single cells isolated from EG7 tumor tissues revealed that I3A treatment led to a significant rise in the frequency of tumor‐infiltrating CD8^+^ T cells, and the proportion of IFNγ expression on tumor‐infiltrated CD8^+^ T cells (Figure S4G, Supporting Information). Consistently, I3A treatment significantly increased the presence of tumor‐infiltrating CD8^+^ T cells in B16 tumors, concomitant with an elevated level of GZMB expression on these infiltrated cells (Figure S4H, Supporting Information), suggesting immune activation in the tumor microenvironment. We also measured the IFNγ production of the tumor‐infiltrating lymphocytes (TILs) isolated from the EG7 or B16 tumors with I3A or vehicle treatment via IFNγ ELISpot assay, and the result showed that I3A treatment boosted IFNγ production level in the TILs of both tumor models (Figure 4D,E). Importantly, I3A treatment had no inhibitory effect on the tumor growth of EG7 and B16‐OVA tumors established on T cell‐deficient nude mice (Figure 4F,I). Furthermore, CD8^+^ T cell depletion in B6 mice using an anti‐CD8 depletion antibody abolished I3A‐induced EG7 and B16‐OVA tumor inhibition (Figure 4G,H,J,K). Taken together, these results suggested that CD8^+^ T cells are required for the in vivo antitumor activity of I3A.

### 2.5 I3A Downregulates AhR in Tumor Cells to Boost T Cell Activation

Previous studies have reported that the type I interferon and NF‐κB pathways play crucial roles in regulating tumor immunogenicity. Therefore, our investigation aimed to determine the potential relationship between I3A‐induced immunogenicity and these signaling pathways.^[^ ^27^, ^28^ ^]^ Our findings revealed that co‐treatment with an Interferon α/β receptor (IFNAR) blocking antibody or NF‐κB pathway inhibitor BAY11‐7082 did not alter T cell activation by I3A‐treated tumor cells. This suggests that the immunogenicity induced by I3A in tumor cells is not dependent on the type I interferon or NF‐κB pathway (Figure S5A,B, Supporting Information).

**Figure 5:**
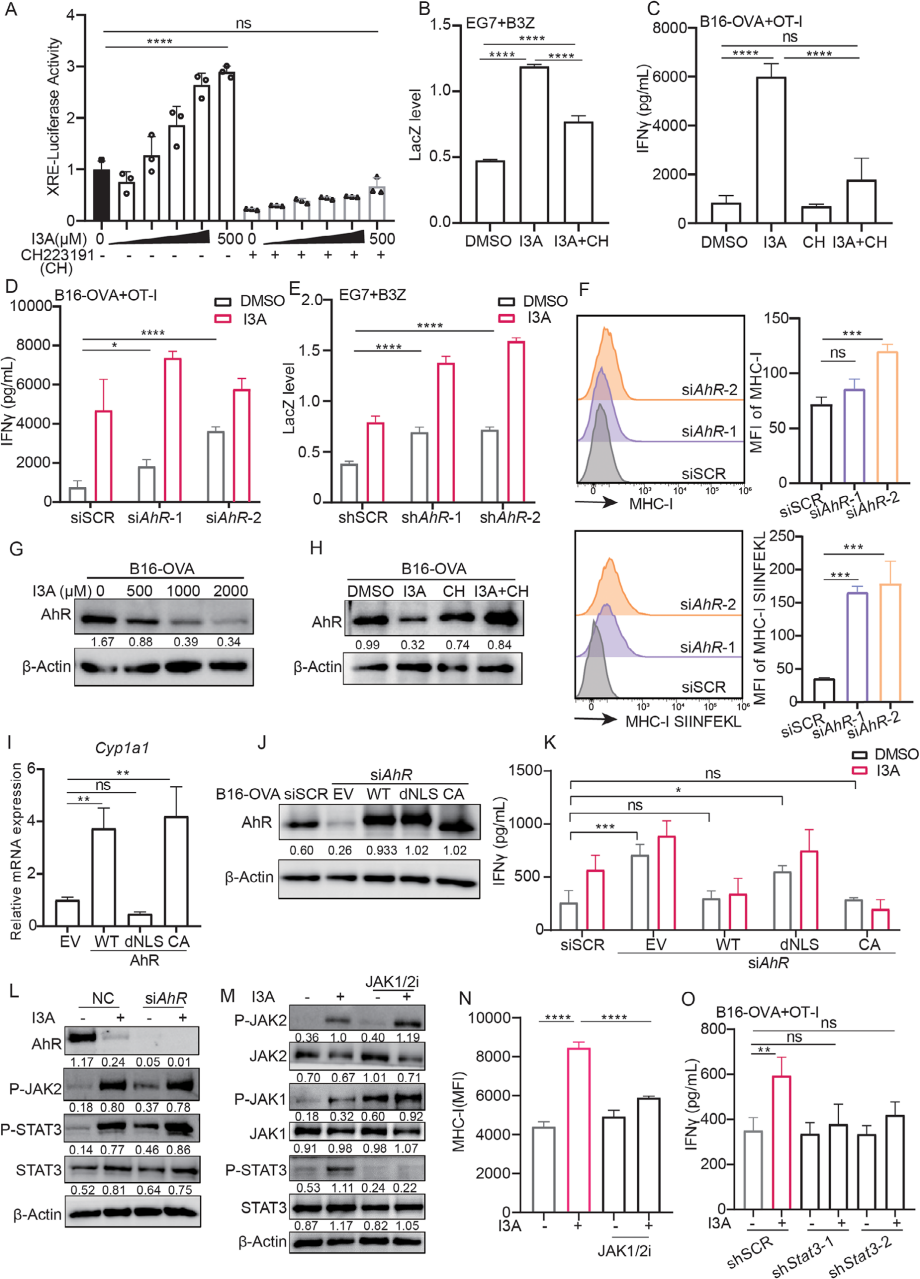
I3A downregulates AhR to boost T cell activation. A) B16‐OVA cells were transfected with XRE‐Luc for 24 h, then treated as indicated for additional 24 h, then the XRE‐luc luciferase activity was measured; CH:CH223191, AhR inhibitor, 50 µm; B,C) EG7 and B16‐OVA cells were treated by I3A or I3A together with AhR inhibitor for 18 h, then co‐cultured with T cells for 24 h, then the IL‐2 promoter activity and IFNγ production level was measured by LacZ assay B) and ELISA C); D) B16‐OVA cells were treated with *AhR*‐targeting siRNAs for 48 h or treated with I3A for 18 h, followed by co‐cultured with naïve OT‐I cells for 24 h, then the supernatant level of IFNγ was measured by ELISA; E) EG7 cells expressing *AhR* shRNA were co‐cultured with B3Z cells for 24 h, then the LacZ activity was measured; F) the expression level of MHC‐I‐SIINFEKL complex and MHC‐I molecule on B16‐OVA expressing si*AhR* were measured by FACS; G,H) B16‐OVA cells were treated with I3A at different concentrations for 18 h, or B16‐OVA cells were treated by I3A or I3A together with AhR inhibitor, then the expression of AhR was detected by Western blot. I) The expression of *Cyp1a1* was measured by q‐PCR assay in B16‐OVA cells expressing AhR‐WT/dNLS/CA mutant; CA: constitutively activated, dNLS: nuclear localization signal deleted. J) Re‐expression of AhR‐WT, AhR‐dNLS, or AhR‐CA mutant protein in B16‐OVA si*AhR* cells was detected by Western blot. K) T cells were co‐cultured with indicated B16‐OVA cells for 18 h, and then the supernatant IFNγ levels were detected by ELISA. L) B16‐OVA cells were treated with *AhR*‐targeting siRNAs for 48 h and treated with I3A for 18 h, then the expression of proteins was detected by Western blot. M) B16‐OVA cells were treated with I3A and JAK inhibitor for 18 h, then the expression levels of proteins were detected by WB. JAK1/2 inhibitor: Ruxolitinib. N) EG7 cells were treated with I3A and JAK inhibitor for 18 h, then the expression levels of MHC‐I were detected by FACS. O) The sh*Stat3* B16‐OVA cells were treated with I3A or not for 18 h, then co‐cultured with naïve OT‐I cells for 24 h, the level of IFNγ was measured by ELISA assay. ns, not significant; Bar graphs represent the average ± SEM. *p*‐Values were derived from unpaired Student's *t*‐test or one‐way ANOVA analysis of variance with Bonferroni's post‐test for panel A‐O. ns, not significant; *p < 0.05; **p < 0.01; ***p < 0.001; ****p < 0.0001. The results shown were representative results of at least 3 biological replicates.

A prior study demonstrated that I3A functions as an aryl hydrocarbon receptor (AhR) agonist, leading to the induction of AhR downstream genes for immune modulation.^[^ ^29^ ^]^ Treatment with I3A resulted in the activation of an XRE‐Luc reporter plasmid, indicating transcriptional activation of AhR by I3A in B16‐OVA tumor cells. Furthermore, the activity was found to be inhibited by the AhR inhibitor CH223191(**Figure** **5**A). Importantly, CH223191 also inhibited I3A‐induced tumor immunogenicity, indicated by lower T cell activation (Figure 5B,C). These results are in line with previous findings that I3A is an AhR agonist, which often induces AhR degradation after activation.^[^ ^30^, ^31^ ^]^

We next tested whether AhR expression in tumor cells could affect T cell activation. Specifically, we utilized siRNA to downregulate AhR in B16‐OVA cells and co‐cultured them with naïve OT‐I T cells. Surprisingly, tumor cells treated with *AhR* siRNA induced higher levels of IFNγ production of T cells (Figure 5D; Figure S6A,B, Supporting Information). Consistently, EG7 cells engineered to express an *AhR*‐specific shRNA exhibited a significant increase in IL‐2 production in T cells (Figure 5E; Figure S6C,D, Supporting Information). B16‐OVA cells pretreated with *AhR* siRNA displayed enhanced surface expression of MHC‐I and MHC‐I‐SIINFEKL complex (Figure 5F).

Activation of the AhR by ligand binding is often followed by the proteasomal degradation of the AhR protein.^[^ ^15^, ^32^, ^33^ ^]^ Indeed, I3A treatment induced a significant decrease in AhR protein level, which was inhibited by proteasome inhibitor MG132, but not autophagy inhibitors (Figure 5G; Figure S6H,I, Supporting Information). Knockdown of *Cul4b*, a known E3 ligase for AhR, also inhibited I3A‐induced AhR degradation in B16‐OVA cells. By contrast, knockdown of *Tiparp*, a factor facilitating nuclear‐cytoplasm translocation of AhR protein for ubiquitination, had no effect on AhR degradation (Figure S6J,K, Supporting Information). Furthermore, I3A‐induced AhR degradation was reversed by the AhR inhibitor CH223191 (Figure 5H; Figure S6E–G, Supporting Information). Hence, it is conceivable that the proteasomal degradation of AhR triggered by its activation, rather than the activation per se, facilitated tumor immunogenicity. To investigate this hypothesis, we expressed AhR‐WT, AhR‐CA (constitutively activated), and AhR‐dNLS (nuclear localization signal deleted) into B16‐OVA cells. We first assessed the mRNA expression levels of the classical AhR target gene *Cyp1a1* in these cells to verify the function of the expressed AhR proteins. We observed the activation of *Cyp1a1* transcription by AhR‐WT and AhR‐CA expression, but not by AhR‐dNLS (Figure 5I), confirming the functional alterations associated with each mutation. Then we expressed WT or mutant AhR into B16‐OVA cells that had been pre‐treated with *AhR*‐specific siRNA, and verified the protein expression by WB (Figure 5J; Figure S6L, Supporting Information). In the tumor‐T co‐culture assay, treatment with *AhR* siRNA resulted in further increase in IFNγ levels of T cells activated by tumor cells in the absence or presence of I3A stimulation. AhR‐WT and AhR‐CA re‐expression in AhR‐knockdown cells lowered I3A‐induced tumor immunogenicity, but AhR‐dNLS did not (Figure 5K). These results suggest that AhR nuclear localization is inhibitory for tumor cell immunogenicity, and I3A may promote degradation of AhR protein to enhance tumor immunogenicity.

We further explored the underlying mechanisms for AhR‐regulated MHC‐I expression by checking the well‐known signaling pathways regulating MHC‐I expression, such as p65, IRF1, IRF2, STAT1, and STAT3.^[^ ^34^ ^]^ The results showed that I3A treatment markedly increased level of phosphorylated JAK2 (p‐JAK2) and phosphorylated STAT3 (p‐STAT3), but not other molecules, and reduced AhR expression induced a higher level of JAK2 phosphorylation, accompanied with increased STAT3 phosphorylation (Figure 5L; Figure S6M, Supporting Information). Both AhR‐WT and AhR‐CA overexpression decreased I3A‐induced p‐STAT3 level, while AhR‐dNLS overexpression failed to inhibit p‐STAT3 upregulation (Figure S6N, Supporting Information). This result is consistent with different effects of each mutant on modulating tumor immunogenicity (Figure 5K). To further investigate whether I3A increased MHC‐I expression via JAK2‐STAT3 signaling axis, we used Ruxolinitib, a JAK1/2 inhibitor, to treat tumor cells together with I3A. The result showed that Ruxolinitib significantly inhibited I3A‐induced p‐STAT3 and the expression of MHC‐I (Figure 5M,N). Further, knockdown of STAT3 by shRNA in B16‐OVA cells markedly inhibited T cell‐secreted IFNγ levels (Figure 5O; Figure S6O, Supporting Information). Collectively, these results suggest that I3A treatment downregulated AhR expression, increased JAK2 and STAT3 phosphorylation, and enhanced the expression of MHC‐I molecules, ultimately improving tumor immunogenicity. Furthermore, tumor cells with reduced AhR expression were found to be more immunogenic, and the use of AhR inhibitors prevented I3A‐induced degradation of AhR and subsequent enhancement of tumor immunogenicity.

### 2.6 I3A Treatment Induces c‐MYC Down‐Regulation in Tumor Cells

In the previous experiment, it was observed that enhanced T cell activation resulting from I3A treatment on B16‐OVA cells could be partially mimicked by AhR knockdown in B16‐OVA cells (Figure 5D). However, I3A treatment of AhR‐knockdown B16‐OVA cells resulted in further enhancement of T cell activation, suggesting the involvement of additional factors beyond AhR for I3A‐induced tumor immunogenicity. To investigate how I3A enhanced tumor immunogenicity apart from AhR downregulation, we performed RNA‐seq analysis to figure out different gene expression patterns in EG7 cells following treatment with I3A. The autophagy pathway was significantly enriched in I3A‐treated cells (Figure S7A–C, Supporting Information). Autophagy has been involved in antigen presentation and chemoresistance of tumor cells mediated by Trp metabolite.^[^ ^35^, ^36^, ^37^ ^]^ Therefore, we investigated the potential relevance of I3A‐induced autophagy to tumor immunogenicity. We first verified that I3A‐treated cells had higher mRNA expression levels of autophagy related genes, and I3A treatment enhanced autophagy flux in protein level, evidenced by increased microtubule‐associated protein light chain 3 beta (LC3B) and P62 protein levels (Figure S7D–F, Supporting Information). Then we treated EG7 tumor cells by I3A together with autophagy inhibitors (CQ, Bafilomycin A1, NH_4_CL, 3MA), followed by co‐cultured with B3Z or OT‐I T cells. However, neither the IL‐2 and IFNγ levels nor the surface expression of CD69 of T cells activated by I3A‐treated tumor cells was changed by autophagy inhibitors (Figure S7G–I, Supporting Information). Furthermore, the IFNγ level in T cells activated by *Atg5* knockout B16‐OVA cells was comparable with that of T cells co‐cultured with sgSCR B16‐OVA cells (Figure S7J–L, Supporting Information). Collectedly, these findings suggest that I3A‐induced tumor immunogenicity was not due to autophagy activation.

**Figure 6:**
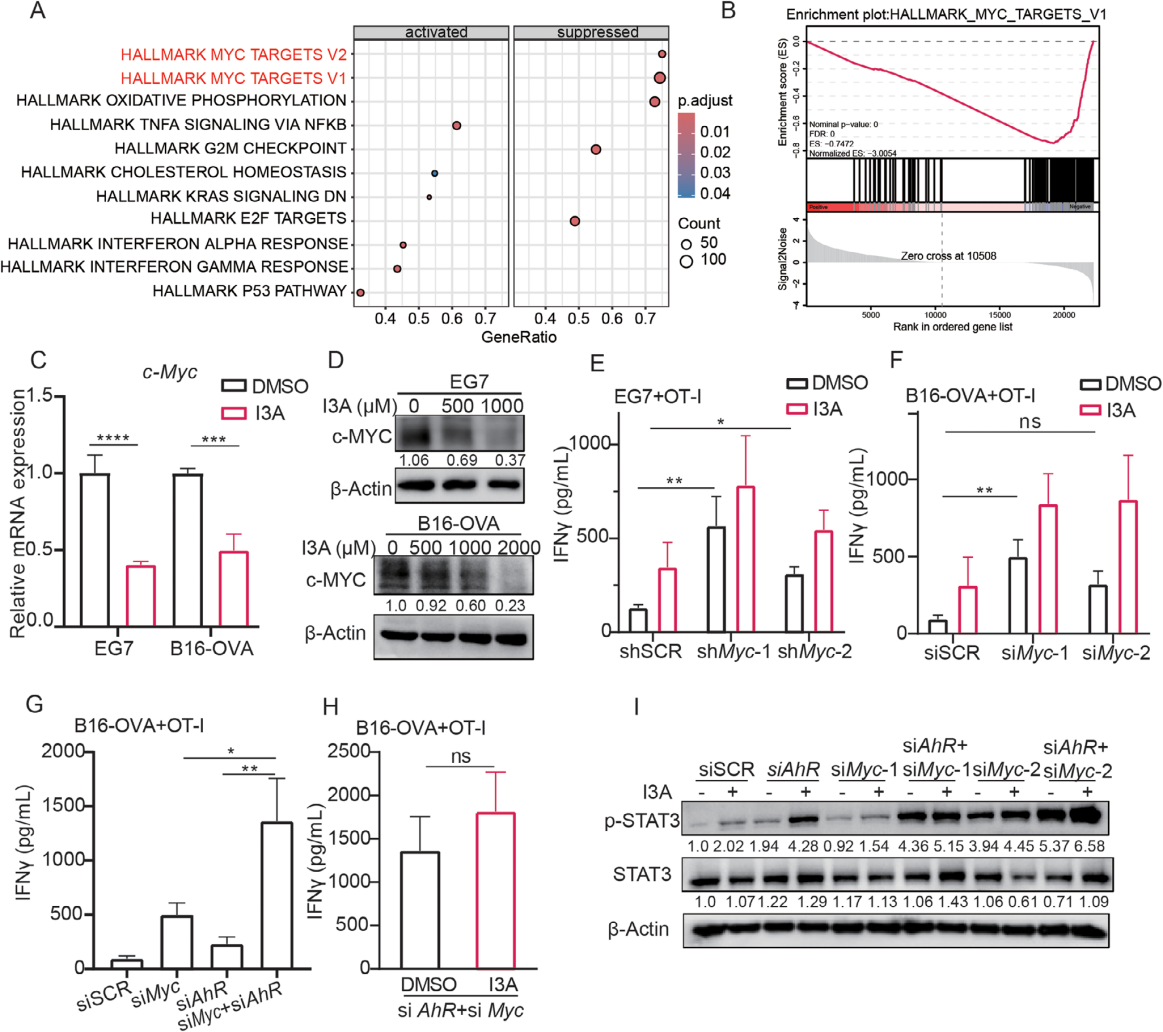
I3A treatment induces c‐MYC degradation in tumor cells. A) Enrichment plots of c‐MYC pathways in EG7 cells which were treated with I3A; B) Gene set enrichment analysis (GSEA) of c‐MYC pathways in EG7 cells treated with I3A; C) B16‐OVA and EG7 cells were treated by I3A for 1 hour and *c‐Myc* mRNA expression were detected by q‐PCR; D) EG7 and B16‐OVA cells were treated by I3A for 18 h and c‐MYC expression were detected by Western blot. E,F) EG7 cells expressing scramble (SCR) or *c‐Myc* targeting shRNAs (sh1, sh2) and B16‐OVA cells were transfected with si*Myc* for 48 h, these cells were treated with I3A or not for 18 h, then co‐cultured with naïve OT‐I cells for 24 h, the level of IFNγ was measured by ELISA assay. G,H) B16‐OVA cells were transfected with different siRNA for 48 h, and co‐cultured with naïve OT‐I T cells for 24 h, then the supernatant levels of IFNγ were measured; I) B16‐OVA cells were transfected with si*AhR* and si*Myc* for 48 h, and treated with I3A for 24 h, the p‐STAT3 and STAT3 protein levels were detected. Bar graphs represent the average ± SEM. *p*‐values were derived from unpaired Student's *t*‐test or one‐way ANOVA analysis of variance with Bonferroni's post‐test for panel C‐H ns, not significant; *p < 0.05; **p < 0.01; ***p < 0.001; ****p < 0.0001. The WB results shown were representative results of at least 3 independent experiments.

Besides autophagy pathway, gene set enrichment analysis (GSEA) of RNA‐seq data also showed a significant enrichment of the c‐MYC relative pathway following I3A treatment (**Figure** **6**A,B). Given previous research indicating that c‐MYC could suppress tumor immunogenicity and promote immune evasion,^[^ ^38^ ^]^ we further verified that I3A treatment significantly reduced *c‐Myc* gene expression by q‐PCR (Figure 6C). Western blot analysis demonstrated that I3A treatment induced c‐MYC protein downregulation in a concentration‐dependent manner (Figure 6D; Figure S8A,B, Supporting Information). Multiple pathways have been found involved in regulation of c‐MYC expression, such as ERK, Wnt‐β‐catenin, TGFβ‐Smad3, and Notch1 pathway.^[^ ^39^ ^]^ We checked the change of these pathways in tumor cells after I3A treatment and found that only the level of phosphorylated ERK (p‐ERK) was significantly decreased upon I3A treatment, and changes of p‐ERK and c‐MYC levels showed similar patterns in tumor cells after I3A treatment (Figure S8C,D, Supporting Information), suggesting that p‐ERK may mediate I3A‐induced c‐MYC downregulation. Meanwhile, co‐culture assay showed c‐MYC knockdown EG7 cells exhibited enhanced IFNγ secretion level of OT‐I cells as compared with that of control tumor cells, and I3A treatment further increased T cell activation levels (Figure 6E; Figure S8E,F, Supporting Information). Similar results were obtained in B16‐OVA cells (Figure 6F; Figure S8E,G, Supporting Information).

We further tested the details for I3A‐induced c‐MYC degradation. Blockade of proteasome but not autophagy inhibited c‐MYC protein degradation (Figure S8H, Supporting Information). Various E3 ligases such as SKP2 and FBW7 mediate c‐MYC ubiquitination and degradation.^[^ ^40^ ^]^ It has been known that T58 phosphorylation of c‐MYC promoted FBW7‐mediated ubiquitination followed by proteasomal degradation, while SKP2 also promoted c‐MYC degradation without the requirement of T58 phosphorylation.^[^ ^41^, ^42^ ^]^ FBW7 depends on T58‐MYC phosphorylation for recognition by SCF^Fbw7^ and then degradation by the 26S proteasome. We then overexpressed either wild‐type c‐MYC or the c‐MYC T58A mutant in 293T cells, then treated the cells with I3A, and found that I3A induced degradation of the WT c‐MYC but not T58A mutant protein (Figure S8I, Supporting Information). Moreover, knocking down of *Fbw7*, but not *Skp2* hindered I3A‐induced c‐MYC degradation (Figure S8J,L, Supporting Information). These results suggested that I3A induces c‐MYC degradation via proteasome pathway depending on the c‐MYC T58 site and the E3 ligase FBW7. Taken together, these results suggest that I3A treatment inhibited p‐ERK, promoted proteasomal degradation of c‐MYC protein in tumor cells, and increased tumor antigen presentation, thereby increasing T cell activation.

Next, we reasoned that AhR and c‐MYC might be the two key factors suppressing tumor immunogenicity, which can be reversed by I3A treatment. Concurrent silencing of *AhR* and *c‐Myc* using siRNA in B16‐OVA cells resulted in the highest level of IFNγ secretion (Figure 6G). Notably, B16‐OVA cells with dual knockdown of *AhR* and *c‐Myc* did not exhibit a further enhancement in IFNγ secretion upon I3A treatment (Figure 6H; Figure S8K,M,N, Supporting Information). Furthermore, dual knockdown of *AhR* and *c‐Myc* achieved the highest level of phosphorylation of STAT3 (Figure 6I). In summary, our results together demonstrated that I3A induces the MHC‐I expression through AhR‐JAK‐STAT3 and ERK‐c‐MYC‐STAT3 pathways on tumor cells to promote tumor immunogenicity.

### 2.7 I3A Sensitizes Tumor Response to Adoptive T Cell Therapy

**Figure 7:**
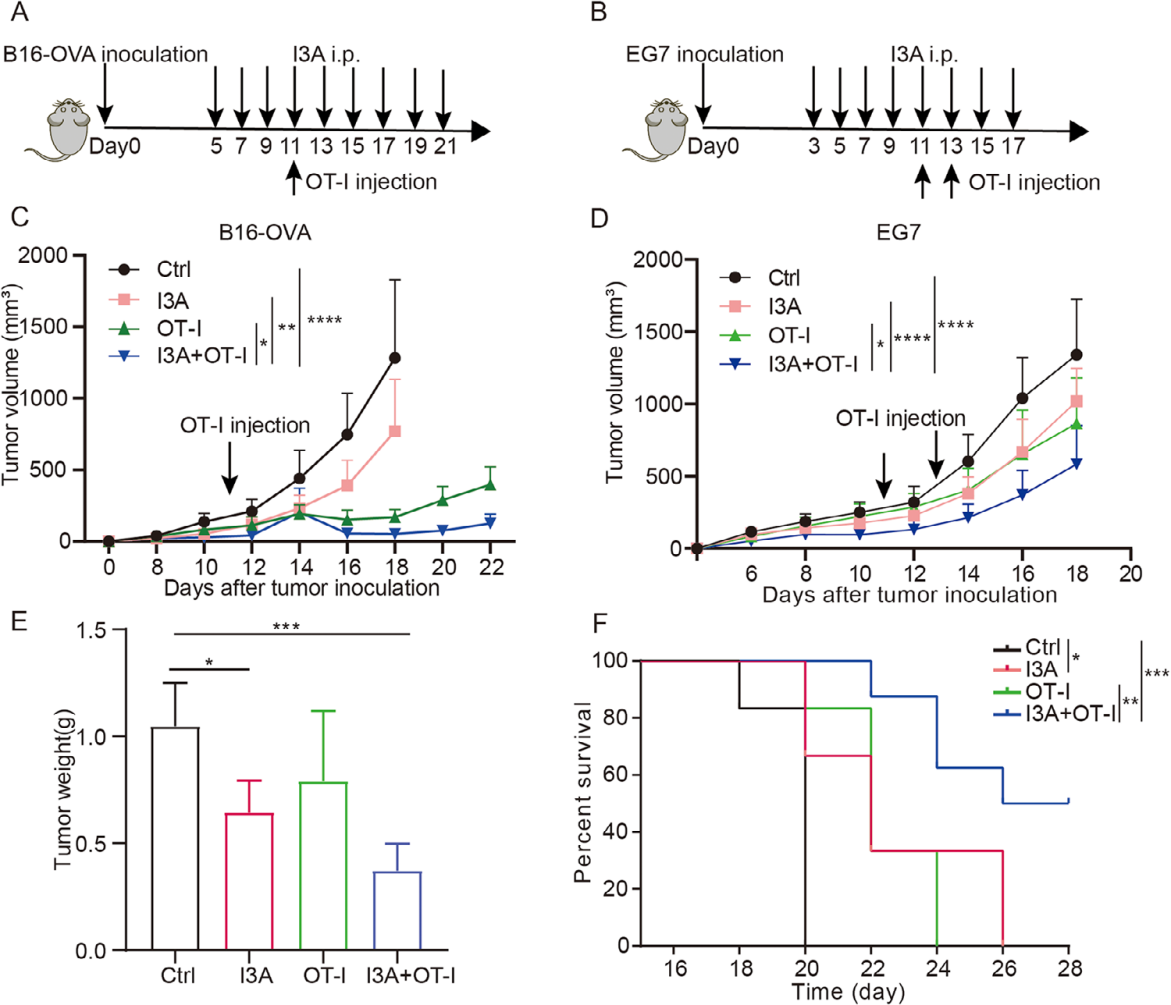
I3A treatment potentiates the antitumor efficacy of adoptive OT‐I T cell therapy. A–D) Schematic illustration of I3A and OT‐I cell transfer treatment plan on mouse melanoma and lymphoma tumor models; B16‐OVA cells (0.2 million per mouse) A,C) and EG7 cells (1 million per mouse) B,D) were s.c. inoculated on B6 mice, then treated with I3A or in combination with activated OT‐I (2 million, tail vein injection), tumor growth was recorded, For B16‐OVA model, n = 5 for Ctrl and I3A+OT‐I groups, and n = 6 for I3A and OT‐I groups; For EG7 model, n = 6 for each group. E) The tumors were isolated from mouse, and tumor weights were recorded. F) Another batch of experiments was performed as in (B), and the Kaplan‐Meier survival curves were plotted and the significance was analyzed by log‐rank test, the endpoint is when the tumor volume reached 2000 mm^3^, n = 6 per group. Bar graphs represent the average ± SEM. *p*‐values were derived from one‐way or two‐way ANOVA analysis of variance with Bonferroni's post‐test for panel C–E. *p < 0.05; ***p < 0.001; ****p < 0.0001.

As I3A significantly enhances tumor immunogenicity, we reasoned that I3A treatment would significantly increase tumor immune recognition and promote anti‐tumor immune response. Therefore, we evaluated the antitumor efficacy of combination therapy using I3A with OT‐I adoptive T cell therapy on B16‐OVA and EG7 tumor models (**Figure** **7**A,B). Our results indicated that I3A treatment or OT‐I T cell transfer alone partially inhibited B16‐OVA and EG7 tumor growth, while the combination of I3A and OT‐I therapy demonstrated the most effective therapeutic effect and prolonged survival (Figure 7C–F). Thus, I3A may sensitize tumors to T cell‐based immunotherapy.

## 3 Discussion

Tumor immunogenicity is not only a predictor of the efficacy of tumor immunotherapy but also an important target to improve the efficacy of tumor immunotherapy. In this study, we found that the Trp metabolite Indole‐3‐aldehyde (I3A) has the ability to augment tumor immunogenicity. More specifically, our findings demonstrate that I3A increases the expression of MHC molecules by inhibiting c‐MYC mRNA expression and facilitating protein degradation, while simultaneously triggering rapid degradation of AhR. Furthermore, I3A exhibited anti‐tumor function in vivo and potentiated the therapeutic efficacy of adoptively transferred T cells. The previous studies reported that *Lactobacillus reuteri* (Lr)‐derived I3A is required for Tc1 cell differentiation in vitro and induces autoimmune hepatitis (AIH)‐like pathology in vivo. Additionally, orally administered *Lactobacillus reuteri (Lr)* migrates to the tumor site and promotes antitumor Tc1 immunity within the tumor.^[^ ^19^, ^43^ ^]^ However, we did not observe a direct T cell activation effect of I3A, likely because we used naïve CD8^+^ T cells, while the previous studies used T cells stimulated with anti‐CD3/anti‐CD28 treatment mimicking T cell receptor (TCR)/co‐stimulation signal.^[^ ^19^, ^43^ ^]^ In conjunction with our findings, we reasoned that I3A may exert its antitumor effect at least in two ways: one by facilitating TCR signaling activation in T cells, and meanwhile inducing tumor immunogenicity. Our study corroborated the diverse impacts of Trp metabolites in the tumor microenvironment, revealing that in addition to the well‐documented immunosuppressive properties of kynurenine, the indole metabolite I3A fosters tumor immunogenicity and bolsters anti‐tumor immune responses. These findings underscore the unique biological effects of Trp metabolites from the kynurenine pathway and the indole pathway.

Previous studies have shown that different AhR agonists have different effects on inducing AhR transcriptional activation and degradation. For example, both TCDD and β‐naphthyl flavone serve as AhR agonists, but the dynamics of nuclear translocation of AhR protein after treatment with the two agonists are not consistent, and the speeds of degradation and restoration of AhR expression in the nucleus are also significantly different.^[^ ^44^ ^]^ In this study, we also found that a variety of Trp metabolites including I3A could transcriptionally activate AhR and induce its degradation, but only I3A could induce tumor immunogenicity, indicating that I3A‐induced AhR translocation and degradation may have a unique downstream effect different from other Trp metabolites, which warrants future investigation. In contrary to our findings, IL4I1 was found to promote neuroblastoma tumor cell motility via I3P production and quick conversion to I3A and KynA facilitated by H_2_O_2_ generated by IL4I1‐catalyzed reaction, while IL4I1 expression in dendritic cells suppresses T cell proliferation.^[^ ^22^, ^45^ ^]^ It is notable that under different conditions, IL4I1‐catalyzed Trp metabolism leads to accumulation of different products, e.g., IAA in 293 cells overexpressing IL4I1, and I3A in in vitro enzyme reaction using purified protein.^[^ ^46^ ^]^ In our co‐culture assay, IL4I1 performed metabolic enzyme function to induce I3A production, which increased tumor immunogenicity. Thus, the expression level of IL4I1 and the intracellular oxidative stress level in different types of cells may lead to different Trp metabolites with distinct immunomodulatory functions, which requires further investigation.

We also demonstrate that I3A enhanced tumor immunogenicity by inducing c‐MYC degradation besides AhR degradation. It has been well established that c‐MYC signaling is often associated with low MHC‐I expression on tumor cells, and c‐MYC downregulation enhances antitumor immune responses via lowering the expression of immunosuppressive molecules including CD47 and PD‐L1.^[^ ^38^, ^39^, ^47^ ^]^ It is thus tempting to speculate a functional or physical interaction between AhR and c‐MYC in regulating MHC‐I expression and tumor immunogenicity. However, individual inhibition of AhR or c‐MYC did not affect the expression of the other protein, and dual knockdown of AhR and c‐MYC in tumor cells induced highest level of T cell activation, suggesting that AhR and c‐MYC downregulation may act in a complimentary rather than interdependent fashion to mediate I3A‐induced tumor immunogenicity. Furthermore, we found that both AhR and c‐MYC inhibition in tumor cells increased level of STAT3 phosphorylation, which was required for I3A‐induced immunogenicity. Previous studies have documented non‐genomic AhR function that can lead to activation of cytoplasmic signaling such as Src, EGFR and JAK pathways.^[^ ^48^, ^49^ ^]^ Our data suggest that I3A activates cytoplasmic JAK/STAT pathway via a non‐genomic AhR signaling. On the other hand, we showed that I3A inhibited p‐ERK and induced c‐MYC protein degradation, which also increased STAT3 phosphorylation and MHC‐I expression. In line with our findings, a recent study reported that an MEK inhibitor upregulated MHC‐I expression depending on STAT3 rather than STAT1 and STAT5.^[^ ^50^ ^]^ Together, these results suggest that both AhR‐JAK and ERK‐c‐MYC axis may together promote I3A‐induced STAT3 phosphorylation and MHC‐I upregulation.

In this study, we investigated the effectiveness of a combination therapy involving indole metabolites and adoptive T cells in the context of tumor immunotherapy. Our findings suggest that this combined approach demonstrates superior efficacy in low immunogenic B16‐OVA melanoma cells. Additionally, we propose a novel strategy of combining immunotherapy with Trp metabolites to enhance treatment outcomes. It was reported that c‐MYC upregulates PD‐L1 expression on tumor cells.^[^ ^38^ ^]^ Therefore, it is tempting to utilize more immunotherapy means such as immune checkpoint blockade antibodies (Anti‐PD‐L1, Anti‐CTLA‐4 antibodies) combining with I3A to improve immunotherapy efficacy. The treatment of immune checkpoint inhibitors is often accompanied by gastrointestinal, endocrine, and dermal toxicity and other adverse reactions. Previous studies have found that I3A can alleviate immune‐associated enteritis, an adverse reaction of immune checkpoint Anti‐CTLA‐4 antibody therapy.^[^ ^51^ ^]^ At the same time, this study also suggested that I3A did not affect the therapeutic effect of Anti‐CTLA‐4 antibody, but we noted that the dose and route of I3A administration in this study (18 mg/kg, p.o.) was different from that of our study (50 mg/kg i.p.). In line with our findings, another study showed synergistic effect of I3A (20 or 40 mg/kg, intratumor injection) and anti‐PD‐L1 therapy on B16 mouse melanoma model.^[^ ^19^ ^]^ Therefore, the therapeutic efficacy of I3A may depend on intratumoral drug concentration, and its combination with immune checkpoint inhibitors needs further study.

Our study found that both I3A and IAA were accumulated in IL4I1‐overexpressing tumor cells. It was recently shown that IAA may suppress autophagy activity of tumor cells through the production of toxic substances by the oxidation of myeloperoxidase derived from neutrophils, thus inhibiting the tolerance of pancreatic cancer to chemotherapy.^[^ ^52^ ^]^ This study suggests that indole metabolites may play an important regulatory role in multiple tumor therapy modalities. Thus, subsequent studies may also explore the immune activation effect of I3A in various tumor therapy contexts such as chemotherapy and radiotherapy.

In conclusion, Trp metabolism has a variety of immunomodulatory effects. The indole metabolite I3A can enhance tumor immunogenicity by targeting c‐MYC and AhR degradation, and exert T cell‐dependent antitumor effect in vivo. Meanwhile, tumor‐intrinsic expression of IL4I1 metabolic enzyme also catalyzed Trp metabolism to I3A and increased tumor immunogenicity. I3A combined with T cell therapy achieved better efficacy in both melanoma and lymphoma models. Our findings connect Trp metabolism with tumor immunogenicity and provide new insights for targeting the indole metabolic branch of Trp for tumor immunotherapy.

## 4 Experimental Section

### Cell Lines

The B16 (C57BL/6J mouse melanoma), and HEK293 cell lines were obtained from ATCC. B16‐OVA cells were constructed as previously reported.^[^ ^6^, ^53^ ^]^ The EL4 (C57BL/6J mouse lymphoma) cell line was kindly gifted by Dr. Wei Yang at Southern Medical University, Guangzhou, China. EG7 cell line was kindly gifted by Dr. Haidong Tang at Tsinghua University, Beijing, China. B3Z hybridoma cells were kindly gifted by Dr. Nilabh Shastri at Johns Hopkins University. All cells were tested as being mycoplasma‐free. The cells were maintained either with Dulbecco's Modified Eagle medium (DMEM Invitrogen) or RPMI‐1640 (Invitrogen) supplemented with 10% FBS and 1% penicillin‐streptomycin in a humidified incubator at 37 °C and 5% CO_2_.

### Primary Cell Culture

CD8^+^ OT‐I T cells were isolated from the spleen and lymph nodes of 8‐week‐old naïve OT‐I mice using MagniSort Mouse CD8 T cell Enrichment Kit (8804‐4622‐74, Thermo Fisher). For in vivo study, OT‐I T cells were cultured in RPMI‐1640 (Gibco, 11875‐176) supplemented with 10% FBS, 1% penicillin‐streptomycin, and 55 µm 2‐mercaptoethanol (Gibco, 21985023) and activated via SIINFEKL peptide (sigma, 10 µg mL^−1^) for 72 h before injection into recipient mice.

### Mice and Reagents

Six to eight‐week‐old female C57BL/6J (B6) mice and nude mice were purchased from the Vital River Laboratory (Beijing, China). OT‐I mice were obtained from Jackson Laboratory. All the mice were maintained under specific pathogen‐free conditions and in accordance with the animal experimental guidelines of Sun Yat‐sen University. All the animal procedures were approved by the Institutional Animal Care and Use Committee of Sun Yat‐sen University.

I3A (Indole‐3‐aldehyde 129445), I3P (Indole‐3‐pyruvic acid I7017), IAA (Indole‐3‐acetic acid I2886), I3C (Indole‐3‐carbinol I7256), 3‐Methylindole (3MI M51458), 3‐Hydroxyanthranilic acid (3HAA 148776), L‐Kynurenine (L‐KYN K8625), 3‐Hydroxy‐DL‐kynurenine (3‐OH‐KYN H1771), Kynurenic acid (KYNA K3375), Anthranilic acid (AA A89855), DMSO (D2650), chlorophenol red β‐D‐galactopyranoside (220588), Tween 80 and PEG300 were purchased from Sigma‐Aldrich. L‐Tryptophan (TRP C6105) was from APEXBIO. CH‐223191(S7711), Ruxolitinib (S1378) were purchased from Selleck. MG132(HY‐13259), Chloroquine (HY‐17589A), Bafilomycin A1(HY‐100558), NH_4_CL(HY‐Y1269), BAY 11–7082 (HY‐13453) and BrdU (HY‐15910) were purchased from MCE.

### LacZ Activity Measurement

The procedures for lacZ activity measurement were performed according to a previously described protocol.^[^ ^5^, ^54^ ^]^ Briefly, after 24 h co‐cultured with pre‐treated tumor cells, B3Z cells in the cell culture plate were lysed by 50 µL LacZ lysis buffer and were freeze‐thawed, then added with 50 µL PBS containing 0.5% bovine serum albumin and 100 µL substrate solution (1 mg/mL chlorophenol red β‐D‐galactopyranoside) dissolved in β‐galactosidase buffer. The plate was incubated at 37 °C for 12 to 16 h until color development reached a proper level, followed by color intensity reading at 590 nm using a microtiter plate reader.

### T Cell Activation Assay and FACS

B16‐OVA or EG7 tumor cells were treated with different metabolites for 18 h, followed by PBS washing to remove metabolites, then tumor cells were resuspended in a suitable volume and co‐cultured with T cells (B3Z or naïve OT‐I cells) at a ratio of 1:1 for indicated time point(s). The LacZ activity in B3Z cells was measured as described. Secreted levels of IL‐2 and IFNγ in supernatant were measured by ELISA kits (88‐7024‐88; 88‐7314‐22, eBioscience). T cells were stained with fluorescence‐labeled antibodies against CD8α (eBioscience,25‐0081‐82), CD69 (Biolegend,104514), CD25 (eBioscience,12‐0251‐82), IFNγ (eBioscience,25‐7311‐82), GZMB (Thermo Fisher, 17‐8898‐82) followed by analysis on flow cytometry (BD‐LSRFortessaTMX‐20).

T cell proliferation was measured by CFSE Cell Division Tracker Kit (423801), the procedure was performed following the kits’ instructions. Briefly, naïve OT‐I T cells were stained by CFSE dye in FBS‐free 1640 medium for 20 min, then the CFSE dye was washed out, and the cells were centrifuged and collected. Metabolite‐treated tumor cells were co‐cultured with CFSE^+^ OT‐I T cells for 72 h, and OT‐I T cell proliferation was detected by FACS.

B16‐OVA or EG7 tumor cells were treated with different metabolites for 18 h or transfected with siRNA for 48 h. Then the washed tumor cells were stained with MHC Class I (H‐2Kb) monoclonal Antibody (eBioscience, AF6‐88.5.5.3(25‐5958‐82)) and OVA_257‐264_ (SIINFEKL) peptide bound to H‐2Kb monoclonal Antibody (eBioscience, eBio25‐D1.16 (25‐D1.16)) at 4 °C for 30 min, then tumor cells were washed by PBS twice and followed by flow cytometry (BD‐LSRFortessaTMX‐20) and analyzed using FlowJo 10.0.

Tumor cell death was measured by the Zombie NIR Fixable Viability Kit (423105), the procedure was performed following the kit's instructions. Briefly, I3A‐treated tumor cells were washed with PBS buffer and resuspended in the 100 µL PBS including Zombie NIR dye (1:1000). Incubate for 10–15 min at RT, protected from light, then the dye was washed out. And the cells were centrifuged and followed by flow cytometry (BD‐LSRFortessaTMX‐20) and analyzed using FlowJo 10.0.

### RNA Extraction, cDNA Synthesis, Quantitative RT‐PCR

Total RNA was isolated using TRIzol (Invitrogen, 15596018) according to the manufacturer's instructions. RNA was reversely transcribed using the HiScript III RT SuperMix for q‐PCR (Vazyme, R323‐01). Real‐time PCR was performed using the SYBR Premix kit (Genstar, A301), and analyzed using the Bio‐Rad CFX96 thermal cycler. The primer sequences used for the investigated mouse genes are shown on Supplement Table S1 (Supporting Information).

### siRNA or shRNA Knockdown

Expression of *AhR/c‐Myc/Stat3* was knocked down by indicated shRNAs from Sigma. Briefly, shRNA‐pLKO.1 vector was co‐transfected with pspaX2 and pMD2.G packaging plasmids in HEK293T cells. The shRNA sequence used was

5′‐GCTCAGGAATTTCCCTACAAA‐3′ for *AhR*‐sh1;

5′‐CATCGACATAACGGACGAAAT‐3′ for *AhR*‐sh2,

 which were cloned into pLKO.1 vector. The shRNA sequence used was

5′‐GCTTCGAAACTCTGGTGCATA‐3′ for *c‐Myc*‐sh1;

5′‐CCTGAAGCAGATCAGCAACAA‐3′ for *c‐Myc*‐sh2,

 which were cloned into pLKO.1 vector. The shRNA sequence used was

5′‐CCTAACTTTGTGGTTCCAGAT‐3′ for *Stat3*‐sh1;

5′‐CCTGAGTTGAATTATCAGCTT‐3′ for *Stat3*‐sh2.

The supernatant was harvested 48 h after transfection and then was used to infect B16‐OVA cells or EG7 cells, followed by puromycin selection for another 3 days. The knockdown effect was assessed by Western blot analysis of whole cell protein extracts. Expression of *AhR/c‐Myc/Stat3* were knocked down by indicated siRNA designed by siDesign center (https://horizondiscovery.com/en/ordering‐and‐calculation‐tools/sidesign‐center) and were synthesized by Ruibiotech. The siRNA was transfected by Lipofectamine RNAiMAX Transfection Reagent (13778150, Invitrogen) according to the manufacturer's instructions. The primers used are listed in Supplementary Table S2 (Supporting Information).

### IL4I1 and AhR cDNA

The full‐length cDNAs of mIL4I1 (Gene ID: 14204) and mAhR (Gene ID:11622) without the termination codon were obtained by polymerase chain reaction. Flag‐IL4I1 overexpression plasmid was established by inserting the IL4I1 cDNA into pCDH‐N‐3 × Flag‐puro vector. Plvx‐AhR overexpression plasmid was established by inserting the AhR cDNA into Plvx‐IRES‐puro vector. A K351A‐mIL4I1 mutant plasmid, mAhR‐CA (ΔPAB), and AhR‐dNLS (RR12/14 AA) plasmid were generated by polymerase chain reaction (PCR) using the KOD‐Neo‐plus (TOYOBO).

### Western Blot

The procedures for protein sample preparation from cell cultures, protein quantification, Western blot, and data analyses were performed as previously described.^[^ ^28^ ^]^ Following antibodies were used for Western blot analyses: β‐ACTIN (Santa Cruz Biotechnology, sc8432), AhR (Enzo‐lifesciences, BML‐SA210‐0100), Flag‐HRP (SIGMA, A8592), c‐MYC (Cell Signaling Technology, 18583), ATG5 (Cell Signaling Technology, 12994), B2M (Abcam, ab75853), Phospho‐STAT3 (Tyr705) (Cell Signaling Technology, 9145), STAT3 (Cell Signaling Technology, 12640), STAT1 (Cell Signaling Technology, 14994), P‐STAT1 (Cell Signaling Technology, 9167), IRF1 (Cell Signaling Technology, 8478), IRF2 (Santa Cruz Biotechnology, sc101069), Phospho‐P65 (Cell Signaling Technology, 3033), P‐ERK (Cell Signaling Technology, 9106), ERK (Cell Signaling Technology, 4695), P‐STAT5 (Cell Signaling Technology, 9351), Cleaved‐NOTCH1 (Cell Signaling Technology, 4147), P‐SMAD3 (Cell Signaling Technology, 9520), β‐CATENIN (Cell Signaling Technology, 8480), P‐JAK1 (Cell Signaling Technology, 74129), P‐JAK2 (Cell Signaling Technology, 8082), JAK2 (Cell Signaling Technology, 3230). Protein bands were visualized by chemiluminescence using an ECL detection kit (NCM Biotech, P10300).

### Metabolites Concentration Measurement

EG7 (5 × 10^6^) cells were treated with I3A for 16 h, or the constructed EV/IL4I1‐WT/IL4I1‐K351A EG7 cells were collected, and the cells were washed with cold PBS twice. Samples were then extracted with LCMS grade methanol (1500 µL per sample), spun for 30 min 4 °C, then centrifuged at 4 °C at 14000 g for 15 min. Collected the supernatant (1300 µL) and dried into powder. The samples were redissolved with 60% acetonitrile solution of 100 µL, shaken for 10 min, centrifuged at 15000 g at 4 °C for 10 min, and the supernatant of 80 µL was added into the injection vial with internal intubation for sample preparation. All samples and standard solutions were detected by UPLC‐ESI‐MS/MS; Agilent 1290 Infinity II liquid phase system and Agilent G6495A mass spectrometry system). Mobile phase A is water (containing 0.1% formic acid) and mobile phase B is acetonitrile (containing 0.1% formic acid). The samples were isolated on Acquity UPLC HSS T3, 1.8 µm, 2.1 mm × 100 mm, Waters column. The target metabolites were gradient elution from 5% to 50% in 10 min with mobile phase B. Mass spectrometry data for each target metabolite were obtained using positive ion mode Multiple reaction monitoring (MRM) parent ions and characteristic product ions. Data processing was performed using MassHunter Quantitation Software (Agilent, B.07.00).

### Tumor Growth, Treatment, and FACS Analysis

For in vivo study, EG7 tumor cells (1 × 10^6^ cells per mouse) and B16‐OVA tumor cells (2 × 10^5^ cells per mouse) were subcutaneously injected into the right flank of B6 or nude mice.

The mice were administrated with I3A or vehicle by intraperitoneal injection (50 mg kg^−1^) from day 3 and day 5 (3%DMSO+30%PEG300+5%TWEEN80). The tumor volume was calculated using the formula 0.5 × tumor length × (tumor width)^2^, where the longer dimension was considered as the tumor length. Anti‐CD8 depletion antibodies (SELLECK, A2102) were i.p. injected on days 3, 6, and 9 (100 µg per mouse) in EG7 tumor and days 4,7, and 10 (100 µg per mouse) in B16‐OVA tumor, and depletion effect was confirmed by flow cytometry.

For immunophenotyping analysis of the tumor microenvironment, EG7 tumor cells, and B16‐OVA tumor cells were subcutaneously injected into the right flank of B6 mice. Then the mice were treated with I3A or vehicle by intraperitoneal injection (50 mg kg^−1^) from day 3 (for EG7 tumors) or day 5 (for B16‐OVA tumors). Tumor tissues were collected and analyzed on day 10 or day 15. For analysis of immune cell populations, mouse tumors were dissociated by gentleMACS (Miltenyi Biotec) and filtered through 70 µm cell strainers to generate single‐cell suspensions, then stained with fluorescence‐labeled antibodies against CD45 (eBioscience, 11‐0451‐82), CD4 (eBioscience, 47‐0041‐82), CD8 (eBioscience, 25‐0081‐82), IFNγ (eBioscience, 25‐7311‐82), Granzyme B (GzmB) (eBioscience, 48‐8898‐82). Fluorescence data were acquired using a BD LSR Fortessa cytometer and analyzed using the FlowJo software, V.7.6.5.

For combination therapy, EG7 tumor cells and B16‐OVA tumor cells were subcutaneously injected into the right flank of B6 mice. Then the mice were randomly divided into four groups; I3A or vehicle was administered by intraperitoneal injection on indicated time points, followed by intravenous injection of OT‐I cells (2 million/mouse) on indicated time points, and the tumor volume was recorded.

### Mouse IFNγ ELISpot Assay

EG7 tumor and B16 tumor cells were subcutaneously injected into the right flank of B6 mice, then the mice were treated with I3A or vehicle as before. Tumor tissues were collected on day 15 and cut into small particles using sterile ophthalmic scissors, and the tumor tissue was transferred to a 200‐mesh cell screen. Grind the tumor tissue by syringe piston until there is no obvious tissue, and rinse the screen with fresh 1640 medium for 2 to 3 times. The lymphocytes were isolated from the single‐cell suspension of tumor tissue by Cytiva Percoll Centrifugation Media (Cytiva 17089101) according to the manufacturer's instructions, centrifuged 550 g for 30 min at 4 °C. The whole procedure should be strictly aseptic.

Tumor infiltrating lymphocytes were seeded in ELISpot plate at 1 × 10^5^ per well with different stimulations. As EG7 tumor model, Tumor infiltrating T cells were stimulated with OVA peptide_257‐264_ at 1 ng µL^−1^. As B16 tumor model, we prepared bone marrow‐derived dendritic cells (BMDCs) in advance. Isolating bone marrow cells from 6‐to‐8‐weeks‐old female WT mice and cultured with 20 ng mL^−1^ GM‐CSF and 20 ng mL^−1^ IL‐4 to induce primary for 5 days. Collected BMDCs at day 5, B16 tumor cells were subjected to three cycles of freezing and thawing. Subsequently, these tumor cells were incubated with mature BMDCs for a duration of 4 h at 1:1 ratio, and the BMDCs feeding the B16 antigen were utilized to stimulate B16 tumor‐infiltrating T cells. Incubation these stimulated cells in ELISpot plate in a 37 °C humidified incubator for 48 h at sterile conditions. Then, the spots were detected according to the manufacturer's instructions (MABTECH, ELISpot Plus: Mouse IFN‐γ (ALP)‐ 3321–4APW‐2). Spot reader data were acquired using an AID ELISpot Reader and analyzed using the AID software.

### Statistics

Data were analyzed using the GraphPad Prism software, 8.0.1. Comparisons between two groups were analyzed using a two‐tailed unpaired Student's *t*‐test. Comparisons between multiple groups were analyzed using one‐way analysis of variance (ANOVA) with Bonferroni's post‐test. Tumor growth was analyzed by two‐way ANOVA with Bonferroni's post‐test. Statistical significance was defined as a P value less than 0.05.

### Study Approval

All mice were maintained under specific pathogen‐free conditions and in accordance with the animal experimental guidelines of Sun Yat‐sen University (Guangzhou, China). All animal procedures were approved by the Institutional Animal Care and Use Committee of Sun Yat‐sen University (Guangzhou, China, Approval # 2022000701).

## Conflict of Interest

The authors declare no conflict of interest.

## Author Contributions

L.C. and Z.W. contributed equally to this work. X.X. conceived and designed the study; L.C. and Z.W. conducted most experiments and drafted the manuscript; Z.G., H.Z., Y.L., H.Z., H.J., F.X., X.W., C.X., Y.L., H.G., and T.W. performed parts of the experiments; Q.Z., P.Z., P.H., J.T., J.B., and J.L. provided reagents and analyzed data; X.X. supervised the project and contributed to manuscript writing.

## Supporting information



Supporting Information

Supporting Information

## Data Availability

The data that support the findings of this study are available from the corresponding author upon reasonable request.;
