# Supplementary material for: Tryptophan Metabolite Indole‐3‐Aldehyde Induces AhR and c‐MYC Degradation to Promote Tumor Immunogenicity
Source: Adv Sci (Weinh). 2025 Jun 29;12(36):e09533. doi: 10.1002/advs.202409533 (PMC12463101; doi:10.1002/advs.202409533)
Supplement: Supplementary file 1 — Supporting Information [file ADVS-12-e09533-s002.docx]

**Supplemental information**

***Figure S1.I3A pre-treated tumor cells induce T cell activation
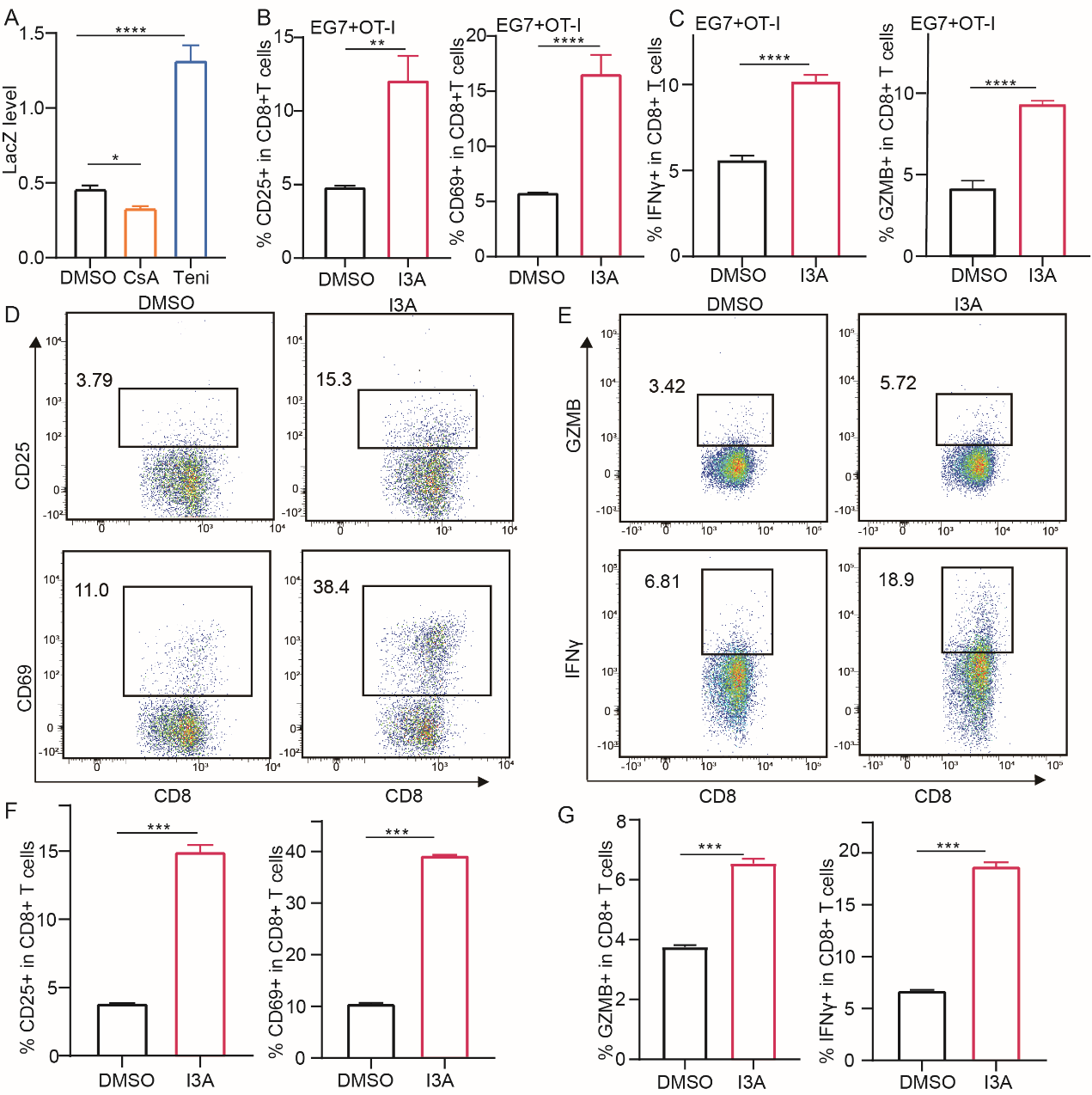
***

**(A)** EG7 cells were treated by Cyclosporine A (CsA, as a negative control) and Teniposide (Teni, as a positive control) for 18 hrs, followed by PBS washing and co-cultured with B3Z T cells to validate screening system. **(B-C)** The percentage of CD25^+^, CD69^+^, IFNγ^+^ and GZMB^+^ OT-I cells after co-culture with I3A-pretreated EG7 cells were quantified after FACS analysis; **(D-E)** B16-OVA cells were pre-treated with I3A (1000 μM) for 18 hrs, followed by PBS washing and co-cultured with naïve OT-I cells for additional 24 hrs. The surface expression of CD25 and CD69, as well as the intracellular expression of IFNγ and GZMB were determined by FACS; **(F-G)** The percentage of CD25^+^, CD69^+^, IFNγ^+^ and GZMB^+^ OT-I cells after co-culture with I3A-pretreated B16-OVA cells were quantified after FACS analysis. Bar graphs represent the average ± SEM. P values were derived from unpaired Student’s t test or one-way ANOVA analysis of variance with Bonferroni’s post-test for panel **A-G**. * *P*<0.05; ** *P*<0.01; *** *P*<0.001; **** *P*<0.0001. Panel **D-E** were representative result of at least 3 independent experiments.

***Figure S2.I3A treatment induces immunogenic markers on tumor cells.***

***
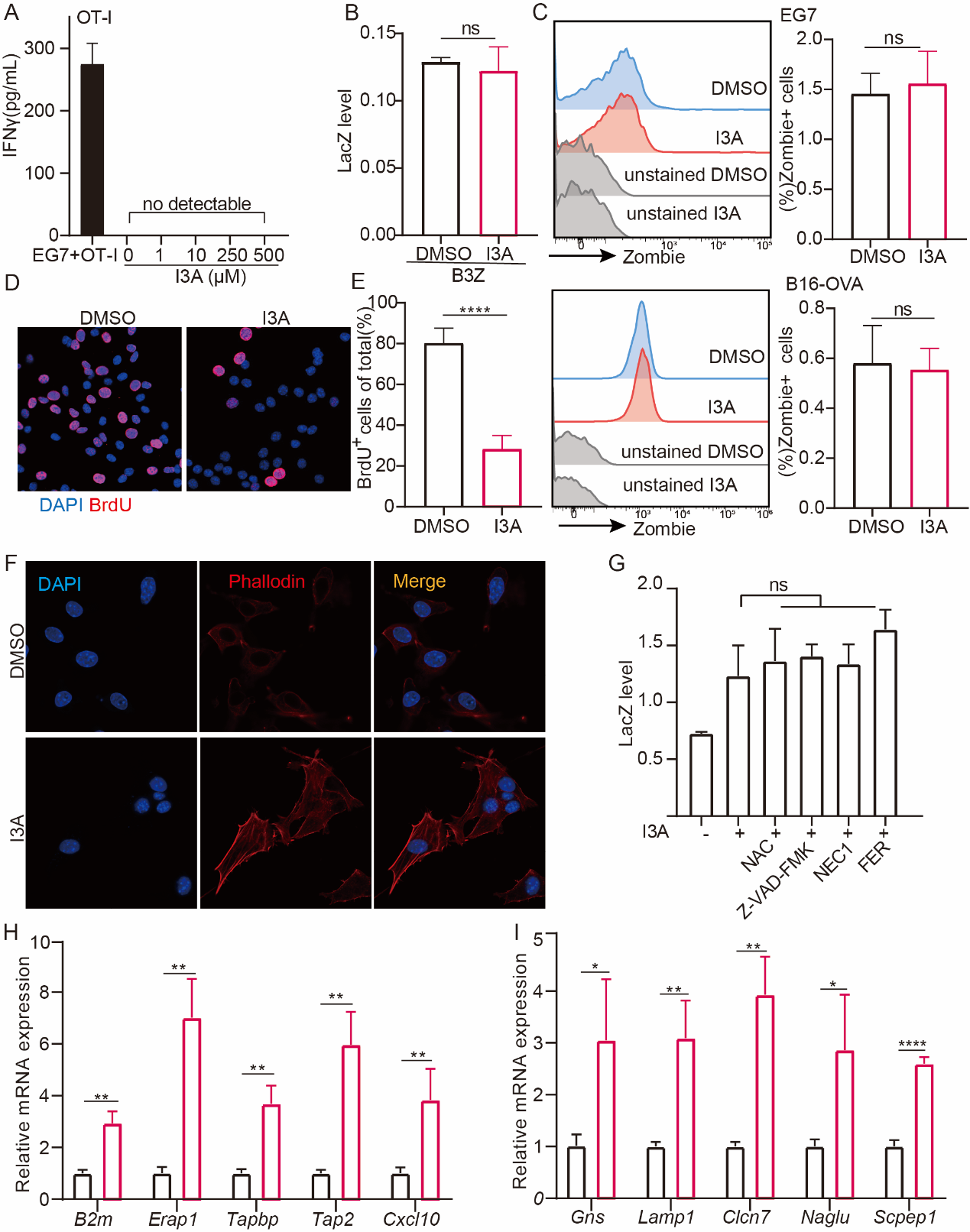
***

**(A)** Naïve OT-I T cells were treated by I3A at different concentration for 18 hrs, then the supernatant IFNγ level was detected by ELISA. EG7+OT-I group means naïve OT-I T cells co-cultured with EG7 cells and used as a positive control. **(B)** B3Z cells were treated with I3A (1000 μM) for 18 hrs, and IL-2 level was detected by LacZ assay. **(C)** EG7 and B16-OVA tumor cells were treated with I3A for 18 hrs, and then the cell death was detected by Zombie dye staining. **(D-E)** B16-OVA tumor cells were treated with I3A for 18 hrs, BrdU was added and cultured for additional 6 hrs, then tumor cells were collected and stained by anti-BrdU antibody for fluorescent microscopy imaging. Representative images demonstrated that I3A inhibited BrdU incorporation in B16-OVA cells. **(F)** Fluorescent staining of cytoskeleton of I3A-treated B16-OVA cells using Rhodamine-Phalloidin; **(G)** EG7 cells were treated by specific cell death pathway inhibitors together with I3A, followed by PBS washing and co-cultured with B3Z T cells, and LacZ activity was detected. NAC (N-Acetyl-L-cysteine): antioxidant; Z-VAD-FMK: pan-caspase inhibitor, apoptosis inhibitor; NEC1 (Necrostatin-1): necroptosis inhibitor; FER (ferrostatin-1): ferroptosis inhibitor. **(H-I)** B16-OVA cells were treated with I3A for 12 hrs, and the expression of lysosome-related genes and antigen-presenting machinery genes were measured by q-PCR. Bar graphs represent the average ± SEM. P values were derived from unpaired Student’s t test or one-way ANOVA analysis of variance with Bonferroni’s post-test for panel **A-I**. ns, not significant; * *P*<0.05; ** *P*<0.01; **** *P*<0.0001. Data shown were representative result of at least 3 independent experiments.

***Figure S3. IL4I1 catalyzes tumor-intrinsic I3A production to induce tumor immunogenicity.***

***
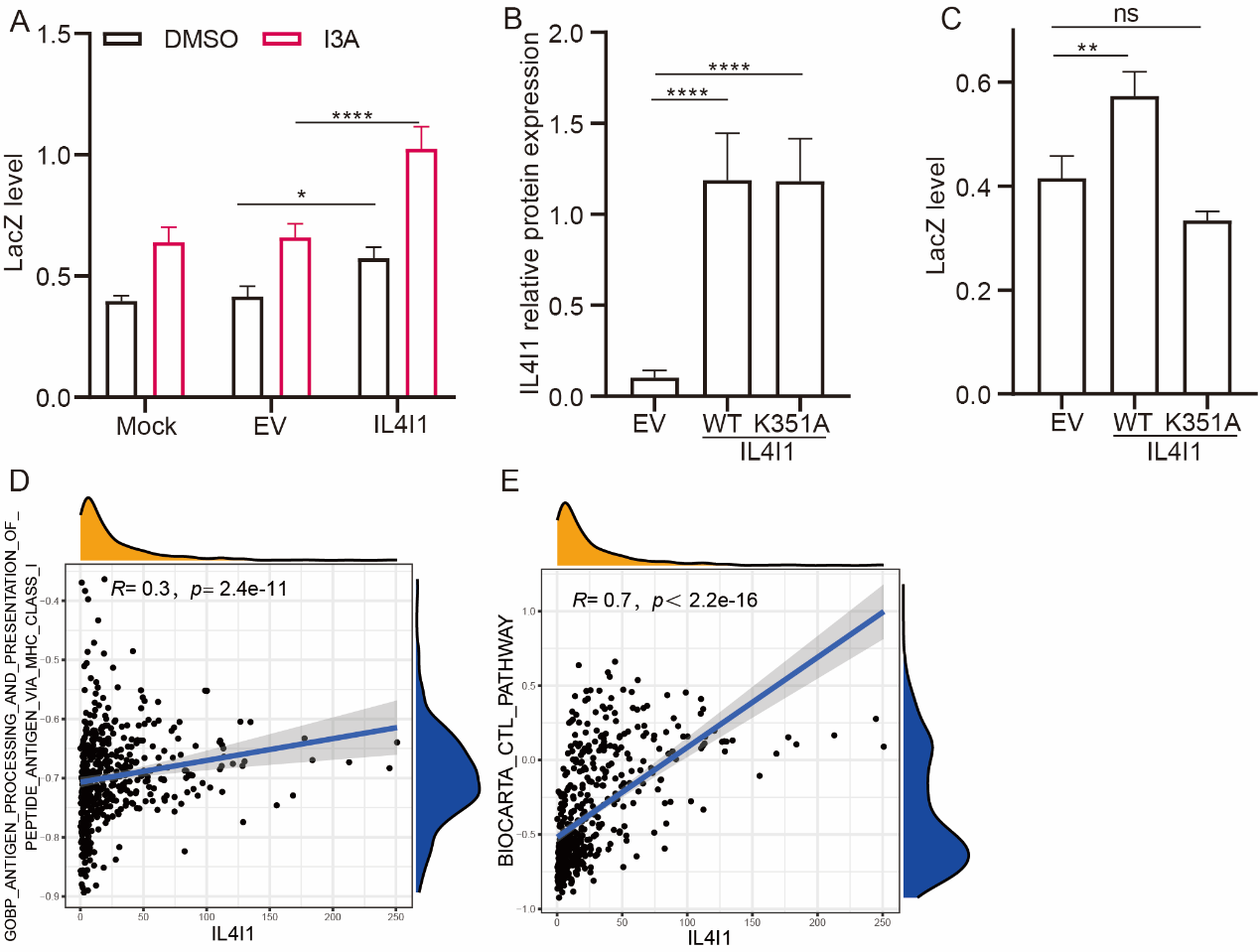
***

**(A)** EG7 tumor cells expressing empty vector (EV) or IL4I1 were treated with I3A for 18 hrs, followed by PBS washing and co-cultured with B3Z T cells for 24 hrs, The LacZ level was measured. Mock: EG7 cells with no treatment. **(B)** The quantified result of the IL4I1 protein expression levels from WB results. **(C)** EG7 tumor cells expressing EV/IL4I1-WT/IL4I1 K351A were co-cultured with B3Z T cells for 24 hrs, then the LacZ level was measured. P values were derived from one-way ANOVA analysis of variance with Bonferroni’s post-test for panel **A-C**. **(D-E)** Correlation of IL4I1 expression with “Antigen Processing and Presentation of Peptide Antigen Via MHC Class I pathway” as well as “CTL activation pathway” in melanoma (SKCM) patients from TCGA database. P values were derived by spearman correlation analysis. Bar graphs represent the average ± SEM. ns, not significant; * *P*<0.05; ** *P*<0.01; **** *P*<0.0001. Data shown in **A-C** were representative result of at least 3 independent experiments.

***Figure S4. I3A inhibits tumor growth via a T cell-dependent manner***


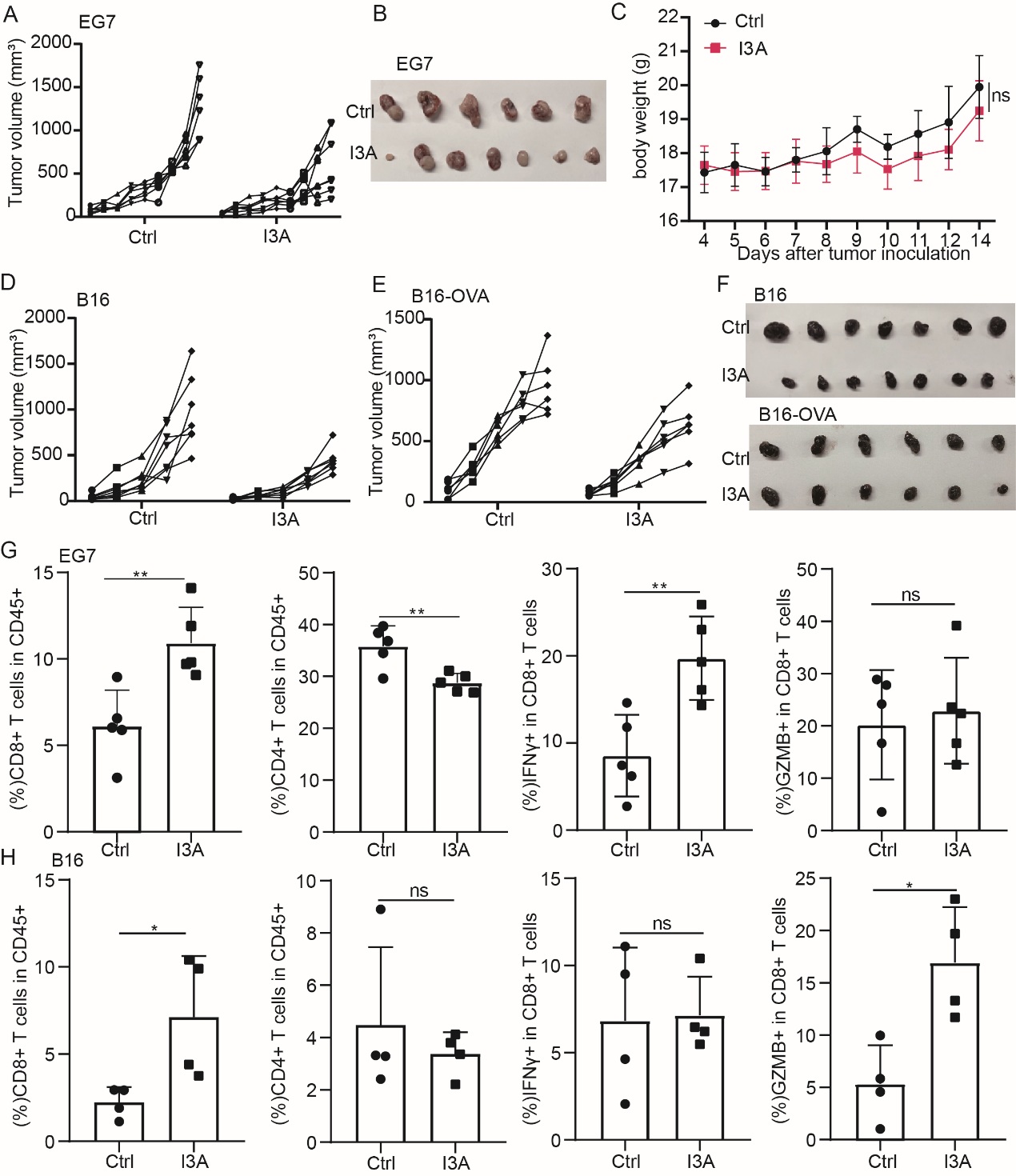


**(A-B)** Individual tumor growth curve of EG7 tumors treated with or without I3A. EG7 tumors were isolated and imaged at the end of experiment. n=7 for I3A group and n=6 for Ctrl group. **(C)** The body weight of C57 mice bearing EG7 tumors was recorded. **(D-F)** Individual tumor growth curve of B16 and B16-OVA tumors treated with or without I3A. Tumors were isolated and imaged at the end of experiment. n=7 for B16 tumor model and n=6 for B16-OVA tumor model. **(G-H)** Mice were injected intraperitoneally (i.p.) with I3A or vehicle from day 3 after EG7 **(G)** or day 5 after B16 **(H)** tumor inoculation, and the tumors were harvested on day 10 or day 15 for immunotyping analysis by FACS; n=5 for EG7 tumors, and n=4 for B16 tumors; Bar graphs represent the average ± SEM. P values were derived from unpaired Student’s t test or two-way ANOVA analysis of variance with Bonferroni’s post-test for panel **A-H**. ns, not significant; * *P*<0.05; ** *P*<0.01.

***Figure S5.I3A-induced tumor immunogenicity is independent on IFN-I or*** ***NF-κB pathway.***


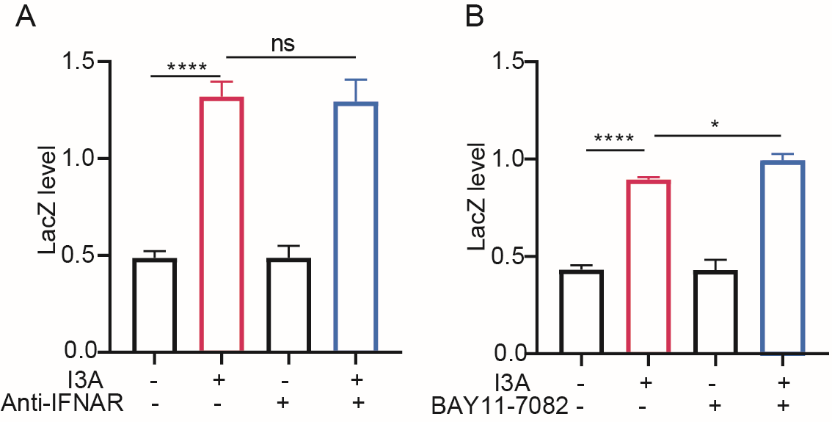


**(A-B)** EG7 cells were treated by I3A together with Interferon α/β receptor (IFNAR) blocking antibody (5 μg/mL) or NF-κB inhibitor BAY11-7082 (5 μM) for 18 hrs, followed by PBS washing and co-cultured with B3Z T cells for additional 24 hrs, then the LacZ activity was measured. Bar graphs represent the average ± SEM. P values were derived from one-way ANOVA analysis of variance with Bonferroni’s post-test for panel **A-B**. ns, not significant; * *P*<0.05; **** *P*<0.0001. The results shown were representative result of at least 3 biological replicates.***Figure S6. I3A downregulates AhR to increase T cell activation***


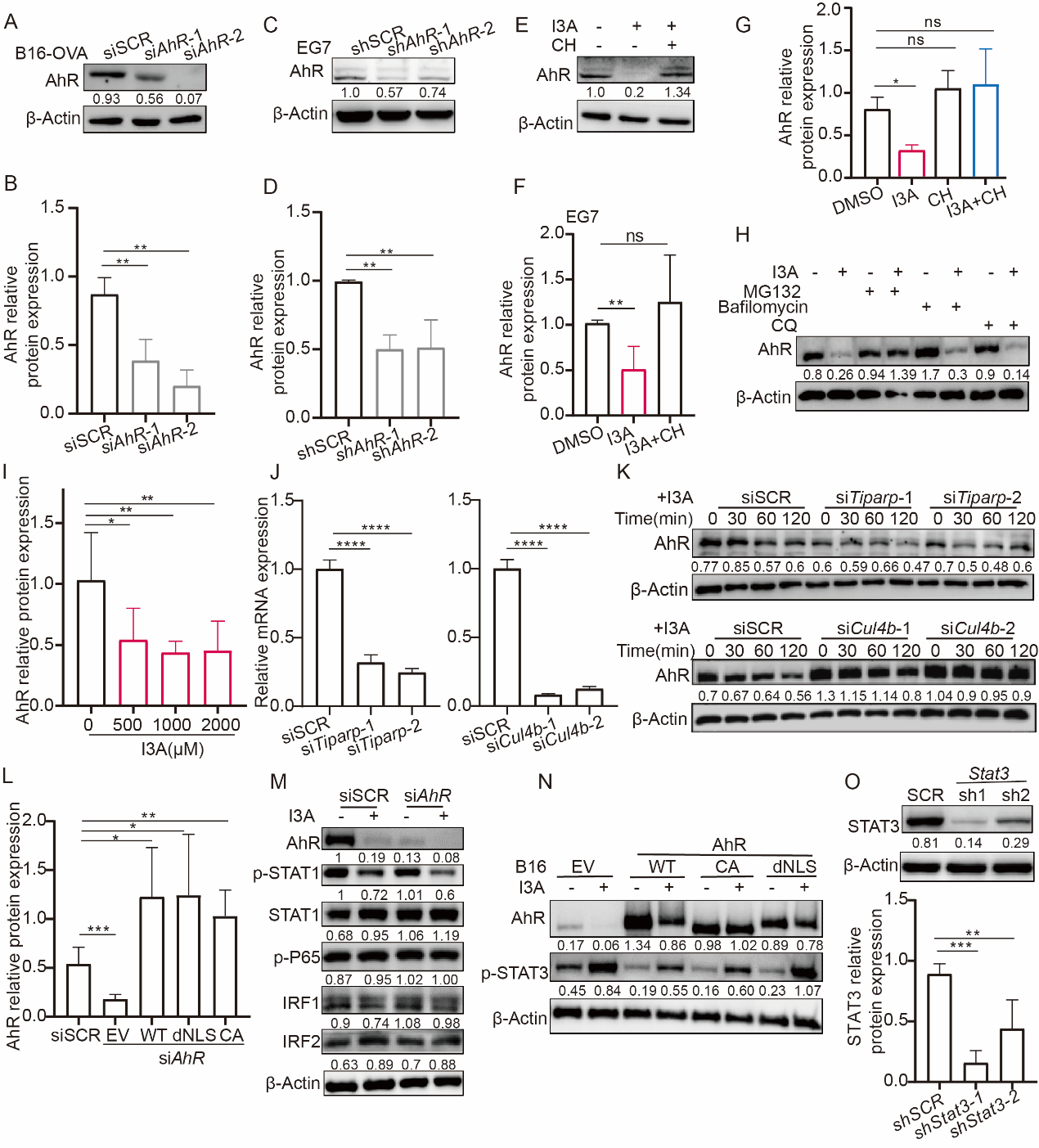


**(A-B)** The expression of AhR following siRNA transfection in B16-OVA cells were measured, and quantitative results were shown in **(B)**; **(C-D)** The knockdown efficiency of *AhR*-targeting shRNAs in EG7 cells were measured, and quantitative results were shown in **(D)**; **(E-F)** Western blot assay showing the expression of AhR in EG7 treated by I3A or I3A + AhR inhibitor (CH223191), and quantitative results were shown in **(F)**; **(G, I)** The protein expression level of AhR in B16-OVA cells were detected by WB in Figure5G-H and quantitative results was shown. **(H)** B16-OVA cells were treated with I3A and different inhibitors for 18 hrs, then the protein levels of AhR were detected by WB. Proteasome inhibitor: MG132, autophagy inhibitors: Bafilomycin, NH_4_CL and Chloroquine(CQ). **(J-K)** B16-OVA cells were transfected with siRNA targeting *Tiparp* or *Cul4b* for 48 hours, then treated by I3A for indicated time; the knock down effect of siRNA were measured by q-PCR assay in (**J)**, and the expression levels of AhR were detected by WB in **(K)**. **(L)** The protein expression level of AhR in B16-OVA cells were detected by WB in Figure5J and quantitative results was shown. **(M)** B16-OVA cells expressing scrambled siRNA (siSCR) or AhR-targeting siRNA (si*AhR*) were treated with I3A for 18 hrs, then the expression levels of different proteins as indicated were detected by WB; **(N)** B16 cells expressing empty vector (EV) or different AhR mutants were treated with I3A for 24 hrs, then the expression levels of p-STAT3 were detected by WB. **(O)** The knockdown efficiency of *Stat3*-targeting shRNAs in B16-OVA cells were measured by WB, and quantitative results were shown. n=3 for each group. Bar graphs represent the average ± SEM. P values were derived from one-way ANOVA analysis of variance with Bonferroni’s post-test for panel **B-O**. ns, not significant; * *P*<0.05; ** *P*<0.01; *** *P*<0.001; **** *P*<0.0001. The results shown were representative result of at least 3 biological replicates.***Figure S7.I3A induces tumor immunogenicity independent of autophagy.***


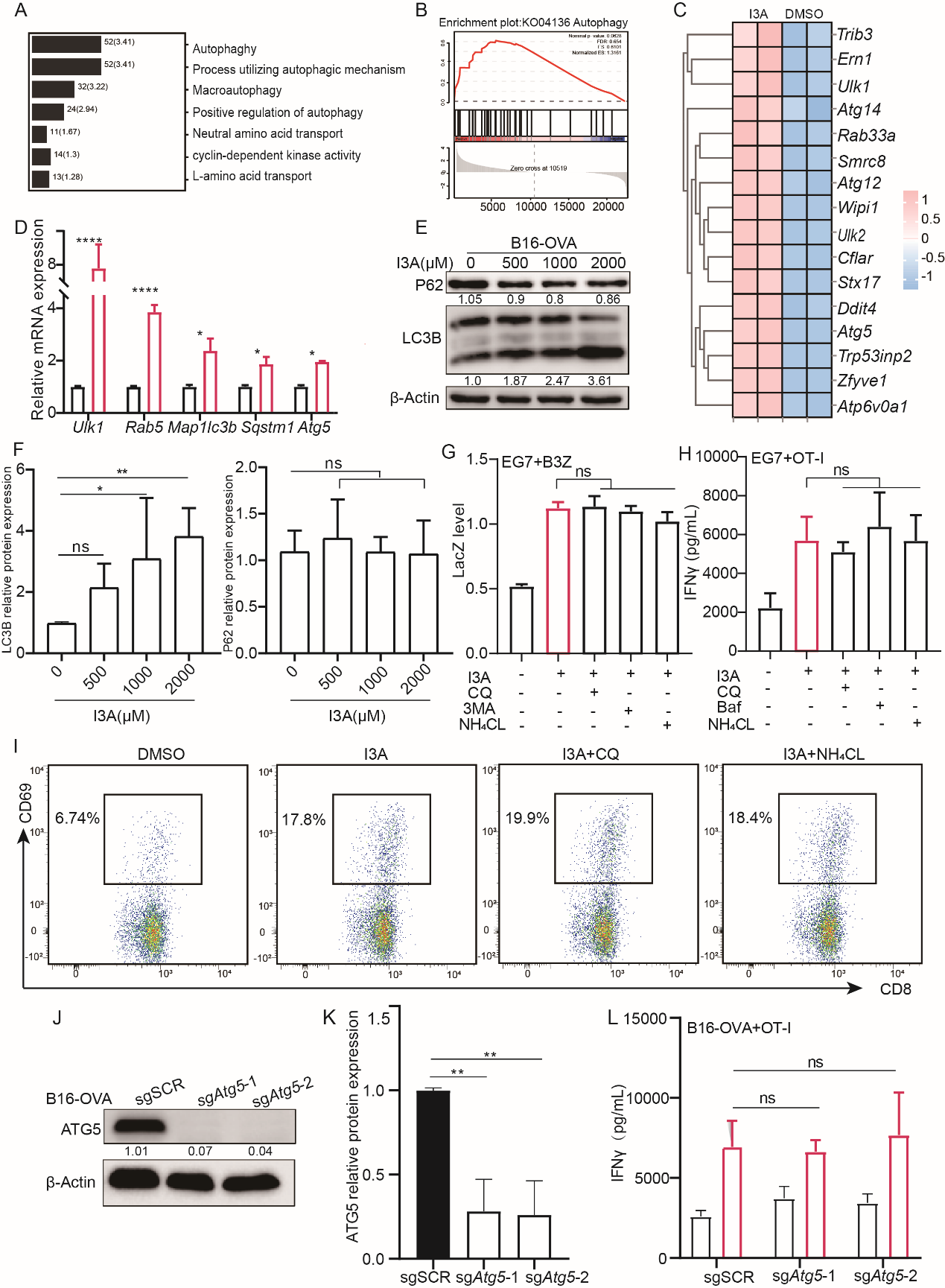


**(A)** Significantly enriched pathways in EG7 cells treated with I3A by RNA-seq; **(B)** Enrichment plots of autophagy pathways in EG7 cells treated with I3A; **(C)** Heat map of gene expression of autophagy pathways in EG7 cells treated with I3A; **(D)** B16-OVA cells were treated by I3A for 12 hrs and autophagy-related gene expression were detected by q-PCR; **(E-F)** Autophagy-related proteins (p62, LC3B) in I3A-treated B16-OVA cells were detected by Western blot. **(G-I)** EG7 tumor cells were treated by I3A together with autophagy pathway inhibitors for 18 hrs, then co-cultured with B3Z **(G)** and OT-I **(H)** T cells, and T cells activation were detected by lacZ assay **(G)** or ELISA assay **(H)**. CQ: Chloroquine; 3-MA:3-Methyladenine; Baf: Bafilomycin A1; NH_4_CL: Ammonium chloride. And the surface expression of CD69 on naïve OT-I T cells was measured by FACS **(I)**. **(J-L)** Western blot detecting the expression of ATG5 on B16-OVA cells **(J-K)**. B16-OVA cells expressing scramble (sgSCR) or *Atg5*-specific sgRNA (sg*Atg5-*1, sg*Atg5-*2) were treated with I3A for 18 hrs, and co-cultured with naïve OT-I T cells for additional 24 hrs, then the supernatant level of IFNγ was measured by ELISA assay **(L)**. ns, not significant. * *P*<0.05; ** *P*<0.01; **** *P*<0.0001. by unpaired Student’s *t* test or one-way ANOVA analysis of variance with Bonferroni’s post-test. Bar graphs represent the average ± SEM. Panel **D-L** were representative result of at least 3 independent experiments.

***Figure S8. I3A downregulates c-MYC in tumor cells to activate T cells.***


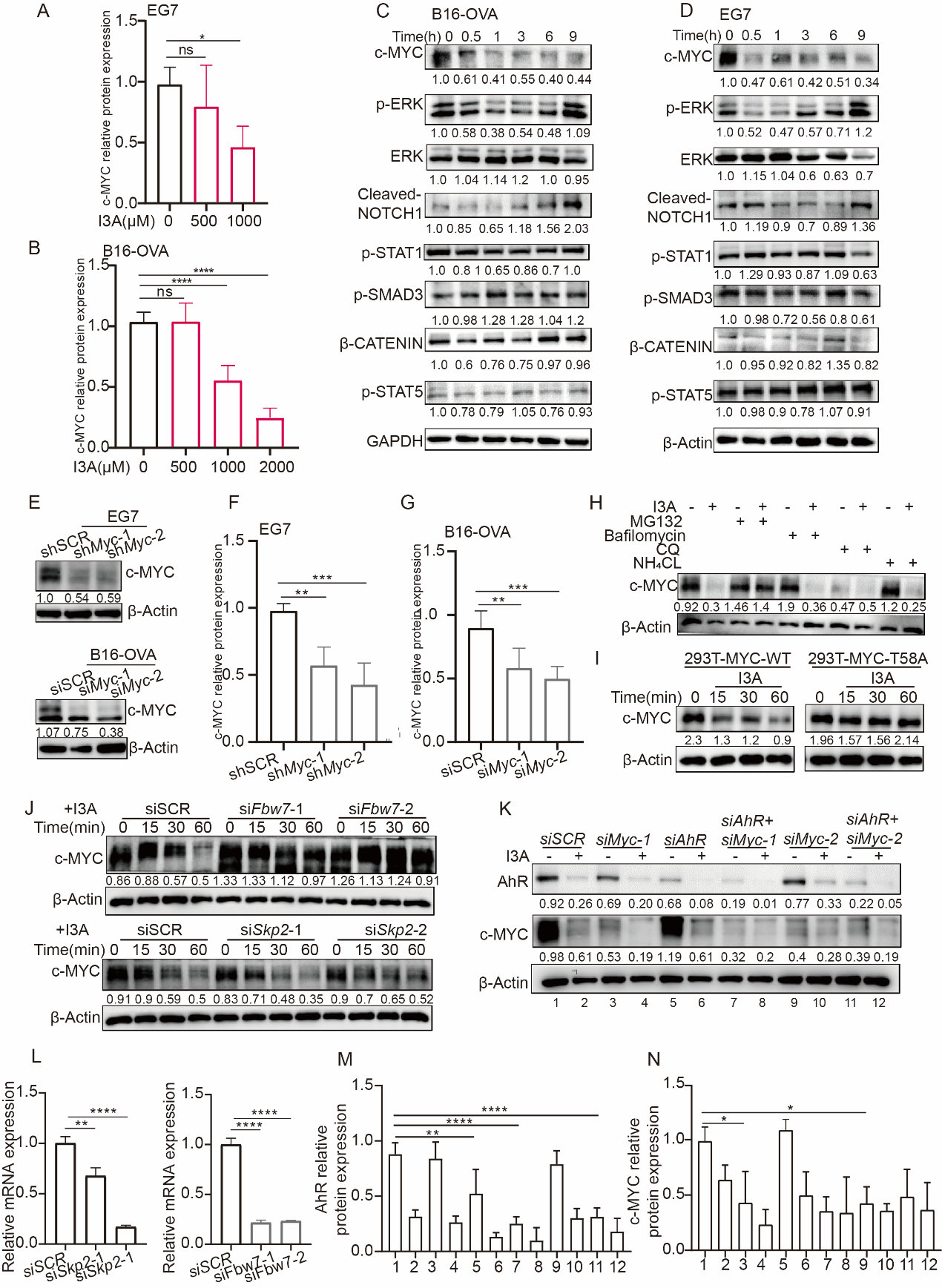


**(A-B)** The expression of c-MYC following I3A treatment in B16-OVA and EG7 cells were measured by western blot, and the quantitative results were shown; **(C-D)** B16-OVA and EG7 tumor cells were treated with I3A and cell lysates were collected at indicated time points for WB assay detecting expression levels of proteins indicated. **(E-G)** The protein expression level of *c-Myc* in EG7 cells expressing *c-Myc*-targeting shRNAs and in B16-OVA cells expressing *c-Myc-*targeting siRNAs were measured by WB, and quantitative results were shown in **(F-G)**; **(H)** B16-OVA cells were treated with I3A and different inhibitors for 18 hrs, then the protein levels of c-MYC were detected by WB. Proteasome inhibitor: MG132, autophagy inhibitors: Bafilomycin, NH_4_CL and Chloroquine(CQ). **(I)** 293T cells expressing c-MYC WT or c-MYC T58A mutant were treated with I3A for indicated time, then the expression levels of c-MYC were detected by WB. **(J, L)** B16-OVA cells were transfected with siRNA targeting *Fbw7* or *Skp2* for 48 hours, then treated by I3A for indicated time; the knock down effect of siRNA were measured by q-PCR assay in (**L)**, and the expression levels of AhR were detected by WB in **(J)**. **(K-N)** B16-OVA cells expressing si*Myc*, si*AhR* and si*Myc* + si*AhR* were treated with I3A for 18 hrs, then the expression of AhR and c-MYC were measured by WB, and quantitative results were shown. P values were derived from one-way ANOVA analysis of variance with Bonferroni’s post-test. ns, not significant; * *P*<0.05; ** *P*<0.01; *** *P*<0.001; **** *P*<0.0001. Panel **B-N** were representative result of at least 3 independent experiments.

**Supplementary Table1. Sequences of primers used for qPCR**

| q-pcr gene (mouse) | primer-F | primer-R |
| --- | --- | --- |
| *B2m* | 5'-TTCTGGTGCTTGTCTCACTGA-3' | 5'-CAGTATGTTCGGCTTCCCATTC-3' |
| *Erap1* | 5'-TAATGGAGACTCATTCCCTTGGA-3' | 5'-AAAGTCAGAGTGCTGAGGTTTG-3' |
| *Tapbp* | 5'-GGCCTGTCTAAGAAACCTGCC-3' | 5'-CCACCTTGAAGTATAGCTTTGGG-3' |
| *Tap2* | 5'-CTGGCGGACATGGCTTTACTT-3' | 5'-CTCCCACTTTTAGCAGTCCCC-3' |
| *Tap1* | 5'-GGACTTGCCTTGTTCCGAGAG-3' | 5'-GCTGCCACATAACTGATAGCGA-3' |
| *Cxcl10* | 5'-CCAAGTGCTGCCGTCATTTTC-3' | 5′-GGCTCGCAGGGATGATTTCAA-3′ |
| *Gns* | 5'-CGGTGTGCGGCTATCAGAC-3' | 5'-CAGGGCATACCAGTAACTCCA-3' |
| *Naglu* | 5'-ACCGCTATTACCAGAATGTGTG-3' | 5'-GTGTGCAAGTTACCCATGCG-3' |
| *Lamp1* | 5'-CAGCACTCTTTGAGGTGAAAAAC-3' | 5'-ACGATCTGAGAACCATTCGCA-3' |
| *Clcn7* | 5'-CGCCAGTCTCATTCTGCACT-3' | 5'-GCTTCTCGTTGTGTGGAATCT-3' |
| *Scpep1* | 5'-CTGCTGCTCCTATCGTTCTTAC-3' | 5'-CCTTTCGGACAGTCACATAATCC-3' |
| *c-myc* | 5'-ATGCCCCTCAACGTGAACTTC-3' | 5'-CGCAACATAGGATGGAGAGCA-3' |
| *Il4I1* | 5'-CAGAAGGTGGTAGTGGTTGGT-3' | 5'-TCATCCCGGAAAGTGAAGATA-3' |
| *Ulk1* | 5'-AAGTTCGAGTTCTCTCGCAAG-3' | 5'-CGATGTTTTCGTGCTTTAGTTCC-3' |
| *Actb* | 5'-AGAGGGAAATCGTGCGTGAC-3' | 5'-CAATAGTGATGACCTGGCCGT-3' |
| *Map1lc3b* | 5'-TTATAGAGCGATACAAGGGGGAG-3' | 5'-CGCCGTCTGATTATCTTGATGAG-3' |
| *Atg5* | 5'-GTCAAATAGCTGACTCTTGGCAA-3' | 5'-TGTGCTTCGAGATGTGTGGTT-3' |
| *Sqstm1* | 5'-AGGATGGGGACTTGGTTGC-3' | 5'-TCACAGATCACATTGGGGTGC-3' |
| *Rab5a* | 5'-GCTAATCGAGGAGCAACAAGAC-3' | 5'-CCAGGCTTGATTTGCCAACAG-3' |
| *18S rRNA* | 5'-TTCCGATAACGAACGAGACTCT-3' | 5'-TGGCTGAACGCCACTTGTC-3' |

**Supplementary Table2. Sequences of sgRNAs or siRNAs**

| gene | primer-F | primer-R |
| --- | --- | --- |
| Sg*Atg5*-1 | CACCGCCTCAACCGCATCCTTGGATGG | AAACCCATCCAAGGATGCGGTTGAGGC |
| Sg*Atg5*-2 | CACCGTCCCATCCAGAGCTGCTTGTGG | AAACCCACAAGCAGCTCTGGATGGGAC |
| si*AhR*-1 | GCTCAGGAATTTCCCTACAAA | TTTGTAGGGAAATTCCTGAGC |
| si*AhR*-2 | AGAGCTCTTTCCGGATAATAA | TTATTATCCGGAAAGAGCTCT |
| si*AhR*-UTR3 | GCUGAAGGUUUCCCAGAAATT | UUUCUGGGAAACCUUCAGCTT |
| si*c-Myc*-1 | CAGGAACUAUGACCUCGACUATT | UAGUCGAGGUCAUAGUUCCUGTT |
| si*c-Myc*-2 | GCUUCGAAACUCUGGUGCAUATT | UAUGCACCAGAGUUUCGAAGCTT |
